# A Comprehensive Analytical Review of Polyphenols: Evaluating Neuroprotection in Alzheimer’s Disease

**DOI:** 10.3390/ijms25115906

**Published:** 2024-05-28

**Authors:** David Vicente-Zurdo, Esther Gómez-Mejía, Noelia Rosales-Conrado, María Eugenia León-González

**Affiliations:** 1 Department of Analytical Chemistry, Faculty of Chemistry Sciences, Complutense University of Madrid, 28040 Madrid, Spain; egomez03@ucm.es (E.G.-M.); nrosales@ucm.es (N.R.-C.); 2 Centre for Metabolomics and Bioanalysis (CEMBIO), Department of Chemistry and Biochemistry, Faculty of Pharmacy, Universidad San Pablo-CEU, CEU Universities, Montepríncipe Urbanization, 28660 Boadilla del Monte, Spain

**Keywords:** polyphenols, Alzheimer’s disease, analytical chemistry, amyloid cascade hypothesis, polyphenols extraction, polyphenols characterization, bioanalytical methods, in vitro assays, in vivo assays, chemometrics

## Abstract

Alzheimer’s Disease (AD), a prevalent neurodegenerative disorder, is the primary cause of dementia. Despite significant advancements in neuroscience, a definitive cure or treatment for this debilitating disease remains elusive. A notable characteristic of AD is oxidative stress, which has been identified as a potential therapeutic target. Polyphenols, secondary metabolites of plant origin, have attracted attention due to their potent antioxidant properties. Epidemiological studies suggest a correlation between the consumption of polyphenol-rich foods and the prevention of chronic diseases, including neurodegenerative disorders, which underscores the potential of polyphenols as a therapeutic strategy in AD management. Hence, this comprehensive review focuses on the diverse roles of polyphenols in AD, with a particular emphasis on neuroprotective potential. Scopus, ScienceDirect, and Google Scholar were used as leading databases for study selection, from 2018 to late March 2024. Analytical chemistry serves as a crucial tool for characterizing polyphenols, with a nuanced exploration of their extraction methods from various sources, often employing chemometric techniques for a holistic interpretation of the advances in this field. Moreover, this review examines current in vitro and in vivo research, aiming to enhance the understanding of polyphenols’ role in AD, and providing valuable insights for forthcoming approaches in this context.

## 1. Introduction

Alzheimer’s disease (AD) is a progressive neurodegenerative disorder that is characterized by the deterioration of cognitive functions, primarily affecting memory and executive functions [1]. The pathophysiology of AD is complex and involves the accumulation of amyloid-beta (Aβ) plaques and tau protein tangles, leading to neuronal loss and synaptic dysfunction. The global prevalence of AD is increasing, with an estimated 50 million people affected worldwide, a number expected to triple by 2050 due to the aging population [2].

Recent studies have highlighted the potential role of lifestyle factors, including diet, in modulating the risk and progression of AD. The investigation into natural bioactive compounds has garnered significant attention due to their prospective ability to postpone or possibly avert the onset of severe non-communicable diseases [3]. In accordance with this perspective, polyphenols, which constitute a varied assortment of phytochemicals found in a multitude of fruits, vegetables, and beverages, have been the subject of intensive research and investment due to their potential for neuroprotection [4]. These compounds are thought to exert their beneficial effects through various mechanisms, including antioxidant activity, modulation of signaling pathways, and interactions with gut microbiota [5]. Therefore, their use as neuroprotective agents guarantee both a greater consumer safety and a promotion of well-being [6,7,8].

Polyphenols represent a vast group of over 8000 structurally diverse compounds that can be classified into several families, such as flavonoids, phenolic acids, stilbenes, lignans, and tannins. These compounds are known for their potent antioxidant properties, which stem from their ability to scavenge free radicals and chelate metal ions [9]. Additionally, polyphenols can modulate cell signaling pathways and gene expression, contributing to their anti-inflammatory, anti-carcinogenic, and cardioprotective effects [10].

In the context of AD, specific polyphenols such as resveratrol, curcumin, and catechins have been extensively studied for their ability to inhibit Aβ aggregation, reduce oxidative stress, and improve cognitive function in preclinical models [11].

In this line, the elucidation and delineation of bioactive polyphenols for deployment as functional neuroprotective agents are of utmost significance for subsequent applications [8]. To achieve this, it is essential to conduct a comprehensive screening and delineation of sources rich in bioactive compounds. Furthermore, it is imperative to establish and evaluate their neuroprotective efficacy through both in vitro and in vivo assays. Concurrently, an examination of their stability throughout processing, preservation, and consumption, focusing on their absorption and bioaccessibility, is warranted to ensure their effective utilization [12].

The extraction of polyphenols from plant sources is a crucial step in their characterization and subsequent application in AD research. Traditional extraction methods, such as solid–liquid extraction (SLE) and maceration, are being complemented by innovative techniques that aim to improve yield, purity, and environmental sustainability. These include ultrasound-assisted extraction (UAE), pressurized liquid extraction, enzymatic assisted extraction, and matrix solid-phase dispersion extraction (MSPD), among others [13,14]. Moreover, the employment of agri-food biowaste as sources of polyphenols emerges as a sustainable and innovative circular economy strategy, that not only allows us to obtained polyphenols for underutilized, abundant, and cost-effective resources, but also contributes to a sustainable management of agri-food bio-residues, minimizing their environmental impact [15].

Regarding the characterization of polyphenols, it involves a combination of chromatographic and spectrometric techniques, such as high-performance liquid chromatography (HPLC), gas chromatography (GC), and mass spectrometry (MS), to identify and quantify individual phenolic compounds [16]. Recent advances in analytical technology have enhanced the sensitivity and specificity of these methods, allowing for a more comprehensive understanding of polyphenol profiles in different plant matrices [17]. Likewise, this characterization should encompass not only a mere examination of the phenolic composition of the extract, but also an evaluation of its bioactive attributes, since this comprehensive approach will facilitate the assessment of the extract’s quality and its qualitative and potential applicability.

In a similar vein, the therapeutic potential of polyphenols in AD has been demonstrated in various epidemiological and clinical studies. Longitudinal studies have shown an inverse relationship between polyphenol intake and the risk of developing AD. Clinical trials have explored the effects of polyphenol-rich diets and supplements on cognitive function in individuals with AD, with some studies reporting improvements in memory and executive function [18].

The therapeutic potential of polyphenols in AD has been underscored by a wealth of in vitro and in vivo studies. These experiments are pivotal for elucidating the mechanisms by which polyphenols exert their neuroprotective effects. In vitro studies provide a controlled environment to explore the interactions between polyphenols and proteins implicated in AD, such as amyloid-beta fragment 42 (Aβ_42_), phosphorylated tau (p-tau), acetylcholinesterase (AChE), and butyrylcholinesterase (BChE) [19]. Through bioanalytical techniques, it can be observed how polyphenols influence the aggregation of Aβ_42_ and p-tau, potentially offering insights into their inhibitory effects on plaque formation [20].

Moreover, the neuroprotective effects of polyphenols have been investigated in various cell lines, which serve as models for AD. These studies often focus on the mitigation of cytotoxicity and the antioxidant properties of polyphenols. For instance, polyphenols have been shown to protect neuronal cells from oxidative stress-induced damage by modulating signaling pathways and reducing inflammatory responses [21]. The effectiveness of these compounds is further evaluated through their impact on cell viability, apoptosis, and neuroinflammation [22].

In vivo studies complement in vitro findings by assessing the efficacy of polyphenols in animal models of AD. These experiments have demonstrated that certain polyphenols can cross the blood–brain barrier (BBB), decrease amyloid levels, and reduce plaque formation in the brains of model organisms [23]. Additionally, polyphenols have been observed to modulate neuroinflammation and oxidative stress in vivo, further supporting their role in neuroprotection [24]. The integration of in vitro and in vivo approaches offers a comprehensive understanding of the neuroprotective potential of polyphenols. Such studies are essential for advancing clinical applications and developing effective polyphenol-based treatments for AD.

The mechanisms underlying the neuroprotective effects of polyphenols are multifaceted. They include a reduction in oxidative stress and neuroinflammation, modulation of Aβ and tau pathology, and enhancement of synaptic plasticity and neurogenesis. These findings support the potential of polyphenols as adjunctive agents in the management of AD, although further research is needed to establish optimal dosing and delivery methods.

Last but not least, chemometric tools are essential for the analysis and interpretation of complex data sets generated in polyphenol research. These tools enable the extraction of meaningful information from high-dimensional data, such as spectral or chromatographic profiles [25]. Techniques such as principal component analysis (PCA), partial least squares regression (PLSR), and hierarchical cluster analysis are commonly used to identify patterns, classify samples, and predict outcomes [26].

In AD research, chemometrics can facilitate the identification of polyphenol biomarkers, the optimization of extraction and characterization methods, and the assessment of the efficacy of polyphenol-based interventions [27]. The integration of chemometric analysis with other omics approaches, such as genomics and metabolomics, offers a promising avenue for advancing our understanding of the role of polyphenols in AD and developing precision medicine strategies [28].

**Figure 1:**
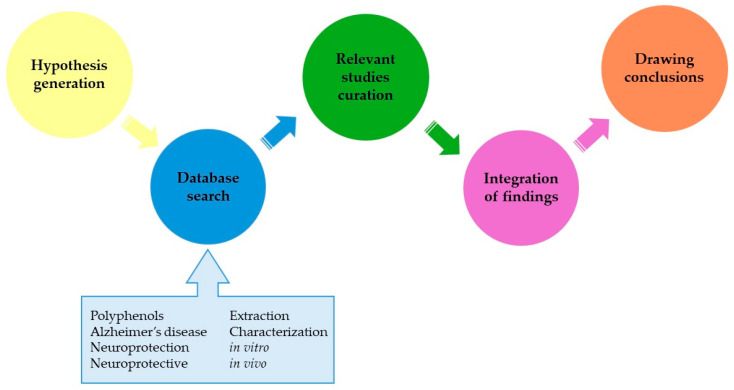
Workflow of the study selection process followed in the preparation of the review.

This review provides a comprehensive recent overview of the current state of research on polyphenols and their potential role in neuroprotection for AD. The selection of studies was primarily conducted using Scopus and ScienceDirect as the foundational databases. Subsequent to the acquisition of principal findings from a systematic search spanning 2018 to late March 2024, Google Scholar was consulted for additional literature. The search strategy implemented the following sequence of keywords: polyphenols AND (Alzheimer’s disease OR neuroprotection OR neuroprotective OR extraction OR characterization OR in vitro OR in vivo). Inclusion criteria were restricted to articles published in the English language. Duplicates were removed to eliminate any redundancies. Moreover, studies that were deemed to offer a substantial contribution to the domain were also included for comprehensive analysis. The workflow followed for the study selection process is presented in Figure 1.

## 2. Polyphenols and Their Obtention

### 2.1. Polyphenols: Characteristics, Sources, and Applications

The term “bioactive compound” denotes naturally occurring molecules, predominantly devoid of nutritional value, which possess the ability to interact with one or more constituents of living tissue, thereby displaying a spectrum of potential health benefits. In other words, it is a biologically active molecule that is inherently present in consumables or other non-edible sources, such as plants, fruits, vegetables, plant-based products (nuts, oils, whole grain…), meat, dairy, mycological entities, and their bio-residues [7,29]. Among the extensive array of structures and functionalities that make up the extraordinary collection of bioactive compounds, polyphenols, also known as phenolic compounds, represent one of the most fascinating, prevalent, and extensively distributed groups of phytochemicals within the *Planate* realm. Yet, it is pertinent to acknowledge that, within this broad spectrum, polyphenols are also prevalent in the fungal domain [29,30].

Phenolic compounds constitute a class of secondary metabolites synthesized mainly by plants, in addition to algae and fungi, serving as a multifaceted defense and signaling tool in the face of diverse abiotic and biotic stressors, including hydric deficits, ultraviolet radiation overexposure, and pathogenic incursions. In addition, empirical evidence from numerous studies substantiates the pivotal function of these phenolic constituents in facilitating the standard ontogenetic processes of plants [31,32]. As such, these compounds are active not solely in imparting the intrinsic chromaticity of vegetation (chief blue, purple, red, and yellow pigments), but also in shaping the organoleptic profiles of fruits and vegetables [31]. Polyphenol synthesis is a complex process involving multiple pathways, including those of shikimic and malonic acid, which account for most plant phenolic compounds, while the latter is rather important in bacteria and fungi. These pathways transform simple carbohydrates into aromatic amino acids, such as tyrosine and phenylalanine, which serve as precursors of phenylpropanoids [29]. Chemically speaking, such secondary metabolites are characterized by presenting a hydrocarbon structure with one or more aromatic rings attached to at least one hydroxyl group [29,30]. This basic backbone can manifest a large number of diverse variations, from simple molecules such as gallic acid (GA) to highly polymerized compounds, underlining the heterogeneity and diversity of the phenolic family compounds. Indeed, in 2010, more than 8000 phenolic structures were already reported [33], a number that has strongly risen to the present time.

**Figure 2:**
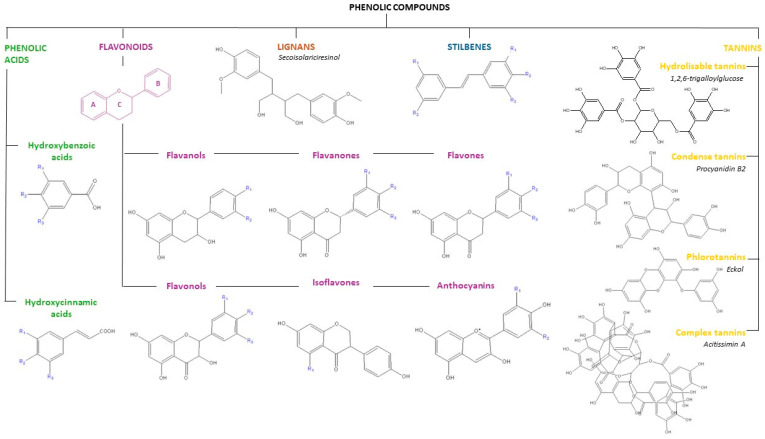
Classification and structure of main phenolic families. The names in italic denote an example of a particular phenolic compound structure within the family. Two phenyl rings (A and B) and a heterocyclic ring (C).

Consequently, the systematization and characterization of polyphenols presents a certain level of complexity, with their origin, biological function, and chemical structure being the most prevalent points [33,34]. Herein, the structural criterion, based on the number of phenolic rings and the structural elements attached to them, is going to be described. Accordingly, as shown in Figure 2, the following main groups can be established: phenolic acids, flavonoids, lignans, stilbenes, and tannins [35,36].

Phenolic acids are characterized by the presence of a carboxylic acid functionality attached to the phenolic ring and can be further classified into hydroxybenzoic and hydroxycinnamic acids, such as GA and ferulic acid, respectively. They typically exist as esters of hydroxy acids, namely quinic, shikimic, and tartaric, and are also derivatives of sugars [29]. Conversely, flavonoids, which constitute the most prevalent group of phenolic compounds, are distinguished by the inclusion of two phenolic rings (rings A and C) connected by a three-carbon oxygenated heterocycle (ring B), as illustrated in Figure 2. The classification of flavonoids into six major subclasses, including flavonols, flavones, isoflavones, flavanols, anthocyanidins, and flavanones, is based on the pattern of hydroxylation and variations in the heterocyclic ring [29,36]. Meanwhile, lignans represent a category of natural phytoestrogens, which are structurally defined by the amalgamation of two phenylpropanoid units interconnected at the β and β’ carbon atoms. These compounds may manifest in the form of aglycones and glycosides, with secoisolariciresinol, matairesinol, and pinoresinol being the most prevalent dietary lignans [29,33]. As for stilbenes, they are polyphenols derived from the phenylpropanoid pathway, which are characterized by two phenyl rings connected by a two-carbon methylene bridge (C6-C2-C6) [33,35,36]. Finally, tannins are high molecular weight polyphenols which are divided into four main categories: hydrolyzable tannins (gallotannins and ellagitannins), condensed tannins, phlorotannins, and complex tannins. Gallotannins are tannins consisting of galloyl units or their meta-depsidic derivatives that are linked to various polyol, catechin, or triterpenoid units, while ellagitannins are tannins with at least two C-C coupled galloyl units without a glycosidically linked catechin unit. Condensed tannins, also called proanthocyanindins, are oligomers composed of flavanol nuclei. Phlorotannins, whose common structural base is phloroglucinol, are a class of tannins found in brown algae and complex tannins are a particular group of tannins in which a catechin unit is glycosidically linked to an ellagitannin or gallotannin unit [29,33,36]. Figure 2 illustrates the chemical structures of the foremost relevant phenolic compounds and their classification.

Concerning the sources of phenolic compounds, numerous studies have documented their ubiquitous occurrence, either free or conjugated, across plant tissues, including vegetative structures (such as stems, leaves, and roots) and reproductive organs (flowers and fruits), as well as in algae and fungi [8,14,27,32]. Notably, these compounds are also prevalent in both edible and non-edible products, encompassing vegetables, fruits, nuts, grains, spices, and processed goods (for instance, beer, coffee, chocolate, infusions, wine, etc.) [14,31,34]. Nonetheless, in recent years, there has been increased recognition of food bio-residues as sources of phenolic compounds, notably, horticultural residues, which encompass all the discarded parts of fruits and vegetables during the different stages of the food supply chain [8,37]. Accordingly, polyphenols have already been identified from the main by-products generated in powerful agri-food manufacturing industries, which include apple pomace, banana peel, coffee pulp and spent coffee grain, grape skins, stems and seeds, olive oil mill, onion skin, and rice bran, to name but a few [38]. On top of that, the use and valorization of agri-food bio-residues as polyphenol sources to be transformed into added-value products emerges as a sustainable circular economy strategy, contributing to the minimization of environmental constrains triggered by food waste disposal, as to the attainment of economic and social benefits [37,39,40]. Table 1 summarizes the major groups of bioactive polyphenols and their representative dietary and agri-food residue sources.

The particular nature and functionality of phenolic compounds has captured the attention of nutritionists, researchers, and manufacturers in various industrial sectors. In particular, the focus has been centered on the potent antioxidant properties of polyphenols and their preventive role in various non-communicable diseases related to oxidative stress, such as metabolic syndrome and diabetes, cardiovascular diseases, neurodegenerative disorders, or cancer [14,38]. In such a way, polyphenols serve as potent antioxidants in a multitude of chemical oxidation systems. Their efficacy is primarily attributed to their capacity to regulate the generation of reactive oxygen species (ROS) and reactive nitrogen species (RNS) by scavenging those species, including but not limited to the hydroxyl radical (OH•), hydrogen peroxide (H_2_O_2_), and the nitroxyl anion (NOˉ) [31,33].

Knowingly, the antioxidant capacity of polyphenols is predominantly determined by their structural characteristics, which facilitate the stabilization of the phenoxy radical, achieved through an amalgamation of the extended delocalization of unpaired electrons within the aromatic π-system and the formation of intramolecular hydrogen bonds [31]. Consequently, phenolic compounds act as superior reducing agents and hydrogen donors, effectively neutralizing the aforementioned reactive species. Additionally, polyphenols possess the ability to chelate metals, thereby inhibiting the production of oxidizing species [36,41].

Further to this, other biological activities have been described in numerous in vitro and in vivo assays, providing anti-inflammatory, anti-hypertensive, antibacterial, antiproliferative, and neuroprotective activity, only to mention a few [14,38,42,43]. This has precipitated a myriad of intended utilizations spanning a diverse array of industrial domains, encompassing pharmaceuticals, agri-food, nutraceutical, cosmetics, and textiles. The primary allure of these advancements resides in the employment of phenolic compounds, which are ostensibly functional and unequivocally less belligerent towards both human well-being and the biosphere [29,44].

One of the leading and forefront applications of phenolic compounds is the development of therapeutic drugs to prevent or treat non-communicable diseases, including neurodegenerative disorders, as well as acting as valuable and efficient antibiotics for communicable diseases [44,45]. In the field of nutraceuticals, polyphenols have been recently acknowledged as a type of prebiotic. They interact with gut microbes and the host and alter the microbial metabolite pool [44]. This makes them interesting in the creation of functional food. Functional foods are those that contain one or more bioactive compounds, added through various technological or biological processes. These foods not only fulfill basic nutritional requirements, but also offer specific physiological benefits, enhance physical well-being, and lower the risk of certain diseases [46].

**Figure 3:**
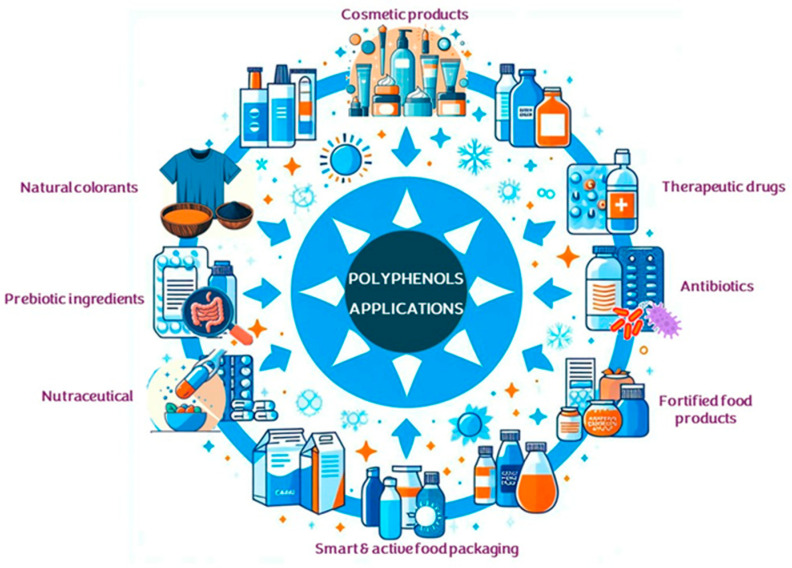
Current and traditional applications of polyphenols in different industrial sectors.

Furthermore, polyphenols have also been exploited for the development of natural dyes, smart and active food packaging, and cosmetic formulations [29,44,47,48]. Figure 3 shows some of the most notable traditional and emerging industrial applications of polyphenols.

Nevertheless, to ascertain the practicality of phenolic compounds as therapeutic agents, it is crucial to consider the metabolic processes and distribution within the bloodstream. These compounds must reach the affected tissues or organs to be effective, while they are subject to mechanical, enzymatic, and metabolic transformations. As a result, such transformations may modify the structure and potential bioactivity of the original phenolic compounds [29]. Hence, the execution of in vitro bioaccessibility assessments, succeeded by in vivo investigations, is imperative in the formulation of polyphenol applications intended for oral or intravenous administration, similar to the scenario with neuroprotective agents.

### 2.2. Extraction Methods for Recovering Polyphenols

For the development of any of the aforementioned applications, particularly those aimed at the prevention and treatment of neurodegenerative diseases, it is imperative to extract polyphenols from the respective biowaste or by-product. Polyphenols are present in a variety of plant-derived materials; however, their quantity and type are contingent upon the extraction methods employed, their chemical nature, the particle size, the presence of interfering compounds, and storage conditions [14]. Consequently, extraction is the pivotal step in procuring phenolic compounds from agri-food matrices, with the objective of merging maximum recovery with the utmost selectivity for the compounds of interest [14,49]. Regrettably, there is no single or standard extraction method, thus, the procedure adopted will ultimately not only determine the type of polyphenols recovered but also the bioactivity of the extract and the potential application thereof.

All things considered, the extraction of phenolic compounds has been accomplished through a variety of techniques, which can be categorized as either conventional or non-conventional [49]. As a result, an exposition of some of the most frequently cited methods in scholarly articles for the extraction of bioactive phenolic compounds from agricultural waste and matrices for the exploration of their neuroprotective potential will be outlined, with an emphasis on their advantages and limitations. Table 2 condenses some of the methodologies developed utilizing the techniques discussed in this context.

#### 2.2.1. Conventional Extraction Techniques

The techniques often referred to as conventional are traditional, well-established extraction methods. These methods depend on the strength of the solvent and/or heat to extract the desired compounds. These have been broadly employed for the extraction of phenolic compounds aiming to study their neuroprotective potential, where the most common are Soxhlet extraction, SLE and maceration [14,40,49].

Soxhlet extraction: this technique involves a series of evaporation and condensation cycles of the solvent, which is poured over the sample and housed in a thimble, resulting in an enrichment of the compounds. This technique was broadly employed for lipid extraction, nevertheless it has been applied for the recovery of phenolic compounds from agricultural waste (see Table 2). Despite its simplicity and potential for automation, it has several drawbacks that have limited its use for phenolic extraction. One of the main issues is the prolonged operation times at elevated temperatures, often exceeding 2 h at 90 °C, which could potentially affect the stability of the phenolic compounds. Additionally, the method requires a significant amount of solvent, typically more than 100 mL, which is another factor that has contributed to its limited use in this context [49,70].

Solid-liquid extraction (SLE): it can be articulated as a mass transfer process wherein analytes, encapsulated within a solid matrix, are translocated into a solvent that is brought into contact with the said matrix [49]. This technique has been extensively utilized for the extraction of polyphenols from a diverse array of agri-food matrices, owing to its manageable operation, cost-effectiveness, user-friendly application at an industrial scale, and efficiency, which is comparable to other non-conventional techniques [49,71] (Table 2). To enhance diffusion and prevent solution saturation near the sample surface, thereby augmenting the speed and yield of the SLE extraction, heating and magnetic or mechanical agitation are often employed in conjunction [72]. In this context, several variables, including temperature, solvent, sample-to-solvent ratio, and extraction time, must be taken into account when optimizing the performance of SLE extraction [36,49]. Nevertheless, it is important to note that SLE-based methodologies do present certain limitations, such as substantial solvent consumption, prolonged extraction times, and significant energy requirements for heating.

Maceration: this traditional technique can be considered a form of SLE, where the ground sample is amalgamated with an extraction solvent, referred to as the menstruum, within a sealed vessel for prolonged durations, typically exceeding 24 h [14,49]. Additionally, the process can be further augmented by agitation, which serves to enhance the yield of the extraction. Despite its time-intensive nature, this cost-effective method has been abundantly applied for the recovery of phenolic compounds with neuroprotective purposes (Table 2).

#### 2.2.2. Non-Conventional Extraction Techniques

The limitations inherent in conventional extraction methods, such as restricted extract purity, prevalent use of costly solvents, and extended extraction durations, have catalyzed the evolution of non-conventional technology-based methodologies [14]. These methodologies are purported to diminish extraction duration and temperature, in addition to solvent consumption, thereby facilitating a more environmentally friendly and energy-efficient recovery of phenolic compounds derived from agri-food bio-residues [49,73]. Hereunder the non-conventional techniques employed for obtaining phenolic extracts for neuroprotective purposes are presented, including UAE, pressurized liquid extraction (PLE), enzyme-assisted extraction (EAE), and MSPD.

Ultrasound-assisted extraction (UAE): the former is predicated on the well-documented phenomenon of cavitation, engendered by the propagation of ultrasound waves at frequencies equal to or surpassing 20 kHz. This propagation engenders longitudinal waves within a liquid medium, modifying expansion and compression cycles and leading to the formation of diminutive gas bubbles that incrementally expand until they collapse, releasing a substantial amount of energy, which instigates significant alterations in pressure and temperature [14]. This considerable energy not only facilitates the rupture of matrix tissues but also augments the mass transfer surface area of the compounds, thereby enhancing extraction efficiency. UAE can be executed with either an ultrasound bath or a focused probe, with the former being more commonly employed due to its universality [74]. UAE-based processes demand lower temperatures and shorter or comparable extraction times to those of conventional SLE methods (Table 2). However, it is not without its shortcomings, including potential sample oxidation due to the formation of free radicals and the critical need to optimize parameters such as ultrasound frequency, power, propagation cycles, and temperature [49,73,74].

Pressurized liquid extraction (PLE): it represents an advanced technique that leverages high temperatures and pressures (103 bar) to maintain the extraction solvent as a subcritical fluid. This condition facilitates a more profound penetration of the solvent into the sample matrix, thereby enhancing the solubility of the target compounds. The elevated temperatures serve to decrease the viscosity and surface tension of the solvent, which in turn accelerates mass transfer [49,73,74]. In terms of operational parameters, the extraction process is executed in cycles, either with a constant volume or solvent flow (static or dynamic mode, respectively), or a combination of both, with the extracts subsequently collected in a vial [75]. This unconventional extraction technology holds significant promise due to its low solvent usage and the prevalent use of water, ethanol, or their combinations [73]. Notwithstanding, it is important to note that this technique necessitates costly equipment and is unsuitable for thermolabile compounds. Furthermore, to optimize the extraction yield, parameters such as the solvent, time, temperature, and number of cycles could be adjusted [49,73].

Enzyme-assisted extraction (EAE): this non-conventional extraction technique takes advantage of the inherent ability of enzymes to degrade cell wall compartments, thus facilitating the migration of the cytoplasmic contents into an extraction fluid such as water [14,49]. The enzymes used are proficient in disrupting and weakening cell walls, as well as in the liberation of bonded polyphenols, which thereby increases the yield of polyphenol extraction. A subset of these enzymes includes cellulases, hemicellulases, and pectinases [14]. Furthermore, it is of significance to highlight that EAE has been acknowledged for its environmental friendliness, attributed to its utilization of water as a solvent in lieu of organic solvents. Nevertheless, its application on an industrial scale is constrained due to the associated costs [14,49]. Along these lines, the beneficial impact of EAE on polyphenol extraction has been poorly documented for the study of phenolic extracts for neuroprotective purposes (Table 2), where different factors, such as enzyme type, molecular size of plant materials, water proportion, pH, and the time are ascribed as key factors for defining the optimal extraction conditions [49,68].

Matrix solid-phase dispersion extraction (MSPD): this advanced technique has been proposed as a more environmentally friendly approach, where extraction and purification of the extract are performed in a single step, increasing sensitivity and selectivity, while shortening protocols [76,77]. Moreover, it has been posited as a straightforward and versatile technique, which has garnered interest for its isolation of compounds prone to degradation, such as phenolic compounds [76,78]. This technique hinges on the grinding of the sample with an abrasive and typically inert material (the dispersant), yielding a semi-dry, homogeneous, and manageable material (the mixture). Moreover, the disruptive dispersant can function as a material, referred to as the sorbent or adjuvant, which can interact with the target analytes and enhance the selectivity and efficiency of this technique [76,78]. Regarding the MSPD extraction procedure, following the grinding of the sample in a mortar equipped with a pestle, the blend is quantitatively transferred to a cartridge furnished with a frit and compressed, whereupon the analytes are then eluted with a suitable solvent [78,79]. Alternatively, ultrasound- or vortex-assisted stirring of the blend–solvent mixture followed by centrifugation can serve as a time-efficient alternative to the conventional SPE elution step [76,79].

#### 2.2.3. Purification of Phenolic Extracts

In both traditional and innovative methodologies, crude extracts often comprise a mixture of different classes of compounds that are soluble in the chosen solvent system. As such, some studies have focused on the removal of unwanted phenolic compounds and other interfering substances, such as proteins, carbohydrates, and waxes, which may require an additional step [14,80].

Therefore, an efficient clean-up method, such as solid phase extraction (SPE), droplet countercurrent chromatography, or column chromatography, is necessary. Furthermore, in recent decades, advances in extraction techniques have sought to amalgamate extraction and sample clean-up in a single step by developing alternative methods for the extraction of specific compounds [14], including the use of molecularly imprinted polymers or that of matrix solid-phase extraction with innovative support materials (e.g., TiO_2_ nanoparticles) [77,81]. These advances aim to streamline the extraction process, reduce the loss of phenolic compounds, and improve the recovery of target analytes [14].

Nevertheless, and contradictorily, isolation and fractionation efforts towards individual polyphenols extracted from agri-food matrices are laborious and may lead to the reduction in their integral activity, where the whole of their constituents are required to observe the synergetic biological effect [82].

### 2.3. Analytical Methods for the Characterization of Phenolic Extracts

Examining polyphenols presents challenges, given the vast array of compounds that may exist in samples and their structural diversity. The complexity is further compounded by the fact that many of these compounds are often present in low concentrations [83]. Consequently, analyzing polyphenols requires sensitive, selective, and precise analytical methods to identify and quantify simultaneously these compounds in complex samples.

Several analytical methods, both spectrophotometric and chromatographic, have been developed and commonly used to determine phenolic compounds in a wide range of samples in the past few decades [36]. Moreover, the integration of sophisticated analytical methods such as HPLC, MS, nuclear magnetic resonance (NMR), and UV-Vis spectroscopy is indispensable for a thorough examination of polyphenols. These techniques play a crucial role in unraveling the intricate polyphenolic profiles present in diverse biological and food matrices, thereby enhancing our comprehension of their significance in health and nutrition [84]. Recent advancements have also highlighted the relevance of high-resolution MS in the targeted and untargeted metabolomic analysis of polyphenols [27].

#### 2.3.1. Spectrophotometric Methods for Quantification of Total Phenolics

Spectrophotometric methods play a pivotal role in the characterization of polyphenol content, providing a versatile, simple and low-cost approach with minimum analytical instrumentation requirements, for quantifying these bioactive compounds. Among the various spectrophotometric techniques, UV-Vis spectroscopy stands out as a popular choice for its simplicity and sensitivity, allowing both rapid screening and quantification of total polyphenol content. These methods rely on the principle that polyphenols exhibit characteristic absorption spectra in the ultraviolet and visible regions due to the presence of conjugated double bonds in their structures, covering a wide range of accepted determinations [36,85].

The absorption maxima are often observed around 280–320 nm, allowing researchers to quantify polyphenols based on their absorbance at specific wavelengths.

**Figure 4:**
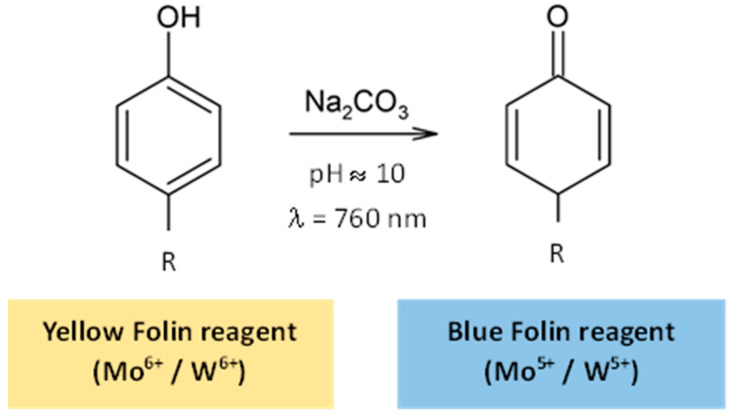
Reaction scheme of the Folin–Ciocalteu method to determine TPC.

Total Phenolic Content (TPC) is frequently determined using the Folin–Ciocalteu colorimetric method, a spectrophotometric assay that measures the reducing capacity of polyphenols. It is based on the redox reaction in alkaline conditions between the functional hydroxy groups of phenolic compounds and the Folin–Ciocalteu reagent, a mixture of phosphotungstic acid (H_3_PW_12_O_40_) and phosphomolybdic acid (H_3_PMo_12_O_40_). This reaction results in the formation of a blue chromophore, whose absorbance is measured at a wavelength around 760 nm (Figure 4). The intensity of the blue chromophore directly corresponds to the concentration of phenolic compounds present in the reaction environment. Therefore, the TPC can be obtained by interpolation on a calibration curve, using GA as standard [85,86,87].

The popularity of this assay can mainly be attributed to its simplicity and speed of analysis, but it presents low specificity, as the color reaction can occur with any oxidizable phenolic hydroxy group, especially if they are present in large amounts [88]. In fact, other substances such as ascorbic acid, aromatic amines, reducing sugars, SO_2_, tyrosine [84,89], vitamins, amino acids, proteins, organic acids [90], and the presence of Fe(III) [86] interfere with the results of the Folin–Ciocalteu assay and they are inadvertently quantified as polyphenols, skewing the results. However, this method continues to be widely applied in food and plant science to measure total phenolics due to its ease of use and low cost.

**Figure 5:**
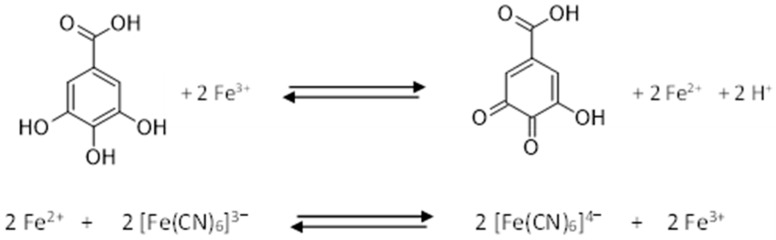
Reactions of the Prussian Blue method.

An alternative spectrophotometric approach for total phenol analysis involves the Prussian Blue method based on the reaction of the phenolic compounds with hexacyanoferrate (III) in the presence of ferric chloride and chlorohydric acid. This results in the formation of hexacyanoferrate (II) ion, [Fe(CN)_6_]^4−^, which further reacts with Fe^3+^, forming a blue metallic complex Fe_4_[Fe(CN)_6_]_3_. The reaction is monitored at 725 nm (Figure 5). While the Prussian Blue assay is less time-intensive and more selective than the Folin–Ciocalteau method, it is susceptible to interference from other compounds. Additionally, the metal complex may precipitate in aqueous conditions, necessitating the adjustment of sample dilution before analysis [91].

Total flavonoids, proanthocyanidin (condensed tannin), and hydrolysable tannin can also be estimated by colorimetric methods [84].

**Figure 6:**
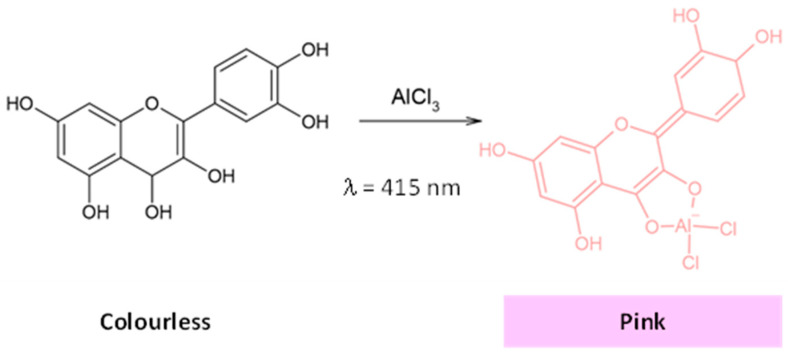
Al(III)-quercetin chelate.

For specific polyphenolic subclasses, such as flavonoids, various spectrophotometric assays are available. The aluminum chloride method, for instance, targets flavonols and flavones, forming a pink-colored flavonoid–aluminum complex that can be quantified at specific wavelengths (410–423 nm) (Figure 6) [92].

Thus, the total flavonoid content (TFC) is determined by a sequence of reactions with AlCl_3_ in alkaline medium (reached by the addition of NaOH) and in the presence of NaNO_2_ decades [36,93]. Subsequently, the TFC can be obtained by interpolation on a calibration curve, using a standard such as quercetin, rutin or catechin [92,93]. This widely used method provides a rapid and cost-effective method for determining the TFC [84]. However, it is remarkable that the reaction time and the chemical organization of the flavonoids can alter significantly the results obtained. Accordingly, the color intensity of the complex depends on the number of available hydroxyl groups, so that flavonoid glycosylated derivatives will not be recognized by this method [92].

The DMAC (*p*-dimethylaminocinnamaldehyde) method has been a longstanding choice for quantifying total flavanols, particularly in fruit juice and beverage samples, where flavanols contribute to sensory properties. This method relies on the interaction between the DMAC reagent and flavanols, leading to a measurable color change detectable through spectrophotometry [89]. However, the accuracy of the DMAC assay to quantify proanthocyanidins is also questionable [84].

**Figure 7:**
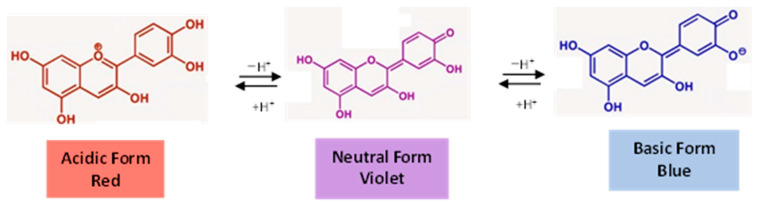
Structural changes of anthocyanin at different pH levels.

Anthocyanins, another main subclass of polyphenols responsible for the vibrant colors in certain fruits and vegetables, can be quantified by spectrophotometric assays using the differential pH method. The process of determining the total monomeric anthocyanins (TMA) relies on the pH-dependent structural changes of anthocyanins. Under highly acidic conditions (pH less than 1), the red flavylium cation is dominant, while the colorless hemiketal form becomes prominent at pH 4.5 (as shown in Figure 7). By assessing the absorbance at the peak wavelength within the red-visible spectrum (approximately 520 nm) in two anthocyanin sample solutions (one dissolved in a KCl solution with a pH of 1 and the other in a sodium acetate buffer with a pH of 4.5), it becomes feasible to quantify the TMA [84]. In addition, the absorbance of each sample solution is also gauged at 700 nm to eliminate any disruption caused by light scattering. The TMA content is then determined taking into account the resulting absorbance, the dilution factor, and the molecular weight and molar extinction coefficient of malvidin-3-O-glucoside, which is considered a standard due to its common occurrence in nature. Lastly, the differential method has been tested and found to be straightforward, quick, and quite precise [36,85].

These spectrophotometric methods offer practical and efficient means for characterizing polyphenols and provide valuable insights into a sample’s polyphenol profile, allowing researchers to quantify and compare levels of polyphenols, flavonoids, and anthocyanins, as well as providing a useful technique for quick and relatively inexpensive screening of numerous samples. However, they do not separate and quantify the compounds individually. In addition, they inevitably have certain errors due to their low specificity and suffer from matrix effects due to the differing solubility of samples, redox potential, and pH differences. Due to these factors, spectrophotometric methods are primarily beneficial for a rough estimation of phenolic compounds. They should be supplemented with more selective and sturdy methods, like chromatographic techniques [36,94].

#### 2.3.2. Chromatographic Methods Used in Separation, Qualitative and Quantitative Polyphenol Analysis

In addition to spectrophotometric approaches that reveal the general polyphenol content, separation techniques present valuable prospects for concurrently profiling and quantifying various bioactive compounds within samples. Within the array of separation techniques employed in contemporary analytical laboratories, chromatographic methods emerge as pivotal tools for the comprehensive characterization of polyphenols, providing powerful tools for the characterization of polyphenols and enabling researchers to separate, quantify, and identify these compounds in diverse biological and food samples. They provide exceptional precision and specificity in both the separation and identification of these intricate compounds.

The choice of chromatographic technique depends on the specific polyphenols of interest and the analytical goals of the study. Among the various chromatographic techniques, GC and liquid chromatography (LC), when coupled with diverse detectors, have been embraced for discerning phenolic compounds in agri-food matrices [95].

GC has been hailed as an effective, precise, and versatile method for polyphenol analysis. However, due to the polar and thermolabile nature of these analytes, direct analysis by GC is hindered, necessitating derivatization (commonly through silylation) to mitigate their polarity. A notable example of its application includes the determination of vanillic and ferulic acid in plum skin and seed residues using GC-MS [96]. Another instance involves the identification and quantification of kaempferol, catechin, chlorogenic acid, and arctigenin in leaves and stems of a wild Nigerian vegetable, achieved through GC coupled to a flame ionization detector [97].

Nevertheless, HPLC emerges as the predominant separation technique for the isolation of polyphenols, owing to its impressive resolution capabilities and remarkable versatility [36,95,98].

HPLC operates in various modes, including reversed phase (RP), normal phase, hydrophilic interaction liquid chromatography (HILIC), or ion exchange chromatography (IEC), among others. These diverse modes offer numerous analytical options for the separation of phenolic compounds from agri-food extracts [95]. Both RP-HPLC and HILIC modes are particularly favored for the analysis of polyphenols, with RP-HPLC being especially preferred. In RP-HPLC, a non-polar stationary phase and a polar hydro-organic mobile phase are employed for separating the compounds. Consequently, the retention of the analyzed compounds increases as the molecules’ hydrophobic nature and the polarity of the mobile phase rise. In contrast, HILIC is designed for hydrophilic compounds, utilizing polar stationary phases and aqueous-organic mobile phases with an organic solvent content exceeding 50%. In contrast to RP-HPLC, HILIC retains the most polar molecules to a greater extent, and these elute as the percentage of water in the mobile phase rises [99].

In RP-HPLC, the separation of phenolic compounds has been effectively achieved using non-polar stationary phases. These are typically based on silica particles that have been modified with long-chain hydrocarbons (C8 and C18) and have sizes ranging from 3 μm to 5 μm. The conventional pore size is between 80 Å and 100 Å. When it comes to mobile phases, binary solvent systems are commonly used. These consist of acidified aqueous solutions and organic solvents such as methanol and acetonitrile. Additionally, acids like formic, acetic, or trifluoroacetic are added to the mobile phase to regulate both pH and ionic strength. This ensures the neutralization of ion-prone polyphenols (for instance, hydroxycinnamic and hydroxybenzoic acids), guaranteeing a much higher and more reproducible retention. Moreover, the chromatographic separation of phenolic extracts is typically carried out in gradient elution mode. However, due to the complexity and diversity of phenolic compounds in agri-food extracts, a prior optimization of the composition and the elution gradient is often necessary [36,95,98,99].

In addition to traditional HPLC, recent years have seen the introduction of advanced LC techniques, including ultrahigh performance liquid chromatography (UHPLC) and capillary liquid chromatography (cLC). These innovations provide a more environmentally friendly option compared to conventional HPLC for analyzing polyphenols in agri-food matrices [95,100]. Revolutionary UHPLC distinguishes itself from traditional HPLC through the utilization of shorter length columns (50 mm) with a smaller inner diameter (ranging between 1 and 2.1 mm), packed with micro particles (under 2 μm). This is complemented by a mobile phase delivery system that operates at ultra-high pressures, reaching up to 1300 bar. This innovative approach yields significant improvements in resolution, peak efficiency, and speed, leading to a reduction in solvent consumption [98]. For example, Azaroual et al. have quantified catechin, epicatechin, caftaric acid, and quercetin derivatives in grape seed residues and skins [101]. Alternatively, cLC represents a miniaturized approach in which separation occurs on columns with a narrower internal diameter (150–500 μm) compared to conventional HPLC columns (3–4.6 mm). Additionally, cLC employs lower flow rates, typically ranging from 1 to 100 μL·min^−1^. The reduction in the internal diameter of chromatographic columns has demonstrated several advantages over traditional HPLC, including shorter analysis times, reduced mobile phase and sample consumption, simplified coupling with MS, and enhanced sensitivity [100]. In the context of analyzing phenolic extracts of agri-food origin, cLC-DAD has been used for the determination of gallic, caffeic, dihydroxy- benzoic and *p*-coumaric acids, catechin, rutin, resveratrol and quercetin in grape residues [77], as well as the major naringin, rutin, and hesperidin in citrus peels [102].

Concerning the detection of polyphenols, a diverse array of detectors has been employed in conjunction with chromatographic separation methods for identifying and quantifying these compounds. UV-Vis spectroscopy, fluorescence, and MS are extensively utilized for characterizing phenolic compounds in agri-food matrices [103].

UV-Vis or diode array detection (DAD) allow for the quantification of individual polyphenolic compounds by measuring their absorbance at specific wavelengths. Present-day multi-way DADs enable the concurrent detection of various phenolic groups. Specifically, the 270–280 nm range is utilized to screen benzoic acids, flavonols, and flavanones. The 310–330 nm range is used for hydroxycinnamic acids and stilbenes. The 370–380 nm range is for flavones, isoflavones, and flavonols. Lastly, the 450–550 nm range is for anthocyanins [95]. However, in LC-DAD determinations the analytes are identified only by retention time and UV spectra. Moreover, it might exhibit other drawbacks, such as lower limits for both detection and quantification in complex samples [103].

Therefore, MS is frequently coupled with HPLC to enhance the identification and structural elucidation of polyphenol-rich extracts. MS enables researchers to detect and characterize these compounds based on their mass-to-charge ratios. It provides information about the molecular mass and fragmentation patterns, aiding in the confirmation of specific polyphenolic compounds. The integration of HPLC with MS offers an abundance of information concerning intricate phenolic mixtures. This combination facilitates the simultaneous screening, confirmation, and quantification of multiple compounds, particularly through tandem mass spectrometry (MS/MS) [95,98]. Various configurations are accessible for these couplings, encompassing ionization sources and single or hybrid mass analyzers such as single quadrupole, ion trap, triple quadrupole, or high-resolution mass analyzers like Orbitrap and time-of-flight (TOF). Nevertheless, electrospray ionization (ESI) stands out as one of the most versatile and widely employed techniques for ionizing phenolic compounds. ESI is a soft ionization method functioning at atmospheric pressure and seamlessly compatible with HPLC. Phenolic compounds can be ionized in both polarities. In the negative ionization mode, although the compounds undergo deprotonation and require higher collision energy for fragmentation, the sensitivity of detection is elevated [95,100,104,105].

LC-MS/MS can enhance noise reduction and sensitivity through the utilization of the multiple reaction monitoring (MRM) scan mode. In recent years, high-resolution mass spectrometry (LC-HRMS) has played an important role in the research of polyphenols, not only for the determination of this family of compounds in food matrices, but also for the characterization and identification of new polyphenols, as well as the classification and authentication of natural extracts in the prevention of frauds [83]. In fact, high-resolution LC-MS and LC–MS/MS techniques, combined with multivariate statistics, have been extensively employed to conduct metabolomic profiling of plant foods for human nutrition. These metabolomics-based approaches, both targeted and untargeted, demand minimal sample preparation and offer a comprehensive insight into the polyphenol composition of the matrix under investigation. This facilitates the assessment of its bioactivity and nutraceutical potential [106].

Another versatile chromatographic technique used to analyze and identify polyphenols is supercritical fluid chromatography (SFC). In contrast to other widely recognized chromatographic methods such as HPLC and GC, SFC boasts superior separation efficiency, high-resolution capabilities, brief analysis times, environmental friendliness, and compatibility with various detectors. This technique has allowed us to successfully identify of eight polyphenols in grape seed extracts [84].

Thin-Layer Chromatography (TLC) is a cost-effective and rapid chromatographic method for preliminary screening of polyphenols. TLC separates compounds on a thin layer of adsorbent material, and the developed chromatogram can be visualized using various staining reagents to identify different polyphenolic classes. Thus, TLC methods have been exploited for the preliminary separation/clean-up of phenolic extracts of virgin olive oil [88], and to separate phenolics in crude plant extracts [84].

#### 2.3.3. Other Analytical Characterization Methods

In addition to chromatographic and spectrophotometric techniques, several non-chromatographic and non-spectrophotometric methods contribute to the comprehensive characterization of polyphenols. These alternative approaches offer complementary approaches for the characterization of polyphenols, providing researchers with a diverse toolkit to explore the chemical and functional aspects of these bioactive compounds.

Electroanalytical methods, and more specifically voltammetric techniques, can be employed to characterize polyphenols based on their redox properties. These methods are particularly useful for studying the antioxidant capacity of polyphenolic compounds, as they allow for the direct measurement of electron transfer reactions. These characteristics allow selective detection of polyphenols with good sensitivity in very complex samples such as wine, being the responses measured independent of the optical path-length or sample turbidity [107].

Additionally, coulometry, an absolute electrochemical method which does not require chemical standards or calibration curves for quantification has been employed for the measurement of antioxidant activities. When a CoulArray detector has been coupled to HPLC, this technique has allowed the determination of several phenolic compounds in food, such as different berry varieties [86].

In recent years, studies based on non-destructive infrared spectroscopy and electrochemical sensors analysis have been performed for tea polyphenol determination. For example, near-infrared spectroscopy was used to quantify the total content polyphenols in green tea, through a fast spectral acquisition and no chemical reagents. A glass carbon electrode and chemically modified electrodes have been also used due to the antioxidant properties of polyphenols for accurate quantification in tea [17]. The advancement of electrochemical biosensors has facilitated the detection of phenolic compounds in the environment. In this context, oxidative enzymes like laccase have been immobilized on the surface of a carbon glass electrode for the purpose of detecting catechol [86].

On the other hand, capillary electrophoresis (CE) is a separation technique that relies on the differential migration of charged polyphenols in an electric field. CE can be coupled with various detection methods, such as UV absorbance, fluorescence, or MS, providing high resolution and efficient separation of polyphenolic compounds based on their electrophoretic mobility. For example, nine polyphenols were determined by CE-ESI-TOFMS from almond-skin extracts, providing information about the presence and relative concentration of minor phenolic compounds [108]. CE has also served as a favorable compromise, balancing analysis time and achieving satisfactory characterization for certain classes of phenolic compounds in virgin olive oil [88].

When mass spectral data fall short of establishing a conclusive structure, NMR spectrometry emerges as a potent complementary method for structural assignment. The NMR spectra of phenolic compounds often exhibit complexity, posing challenges in identifying isolated compounds, particularly when suitable reference standards are absent. This predicament necessitates time-consuming syntheses of relevant materials. While 2D NMR spectrometry can perform structural analysis even in the absence of reference compounds, it demands relatively substantial quantities of the substances. The current drawbacks of NMR spectrometry lie in its limited sensitivity and the requirement to isolate sizable sample quantities. Thus, in numerous cases, the combination of UV, MS, and ^1^H NMR proves sufficient to glean information for structural elucidation [88].

### 2.4. Application of Chemometrics in Polyphenol Characterization

The statistical and chemometric tools used by the researchers are diverse and include the comparison of three or more data sets using one-factor or multi-factor analysis of variance (ANOVA) and various post-hoc methods when there are statistical differences between the mean values of the different groups evaluated. Regarding the study of the relationships between the contents of phenolic compounds and the chemical or biological activities evaluated, the studies focus on correlation analysis, multivariate linear regression or PCA. To optimize the extraction of polyphenols from different natural sources, experimental design has also been used in order to optimize the properties of the extracts with a minimum number of experiments and to know the relationships that exist between the optimized factors and the chemical or biological response measured in each case.

#### 2.4.1. ANOVA and Post-Hoc Methods

ANOVA allows for calculating differences between the means of three or more groups, and when the null hypothesis that all groups have statistically equal mean values at a predetermined level of probability is rejected, it is necessary to perform a multiple comparison test. In many cases, they are called post-hoc tests. Numerous authors do not specify the test used, but the results obtained in the comparison process may be different depending on the test used.

There are several possible classifications of these tests, but one of the most common is to consider a single-step test or stepwise test. In the first case, a type I or α error (false positive) is assumed as a hypothesis and a comparison is made by pairs of values, or in other words, each comparison of pairs of values is independent. Within this group is the Fisher test (least significant difference, LSD), the Tukey test, the Bonferroni test, or the Dunnet test, among others. In all of them, the differences between the values are compared, in pairs, using a single critical value. In the stepwise procedure, type I errors are also considered, and pairwise comparisons are only performed when the results of the previous comparison are statistically significant, within this group is the Duncan test. In this type of test, the critical value of comparison between pairs of means varies for each pair, and if one of the comparisons fails to reject the null hypothesis, all remaining comparisons are rejected.

The most commonly used test is the Tukey test, which is a pairwise post-hoc test, which is designed for the same sample size per level, although Kramer’s modification allows it to be used for unbalanced data. For comparison between the mean values of two pairs of groups, this test uses a *q*-statistic that is generally higher than the Student’s *t*-value and is based on this distribution. The Tukey test has been used to evaluate differences found between different groups in order to evaluate the possible efficacy of specific polyphenols or extracts of polyphenol in animal models that include mice that have been induced for AD with aluminum chloride [59,61], with streptozotocin [109], with scopolamine [110], or in transgenic amyloid precursor protein (APP)/PS1 (Tg) mice [111]. Post-hoc statistical evaluation with this test has also been used in other animal models such as zebrafish that are induced to have memory deficits with scopolamine [64]. The test has been used to evaluate oxidative resistance in *Caenorhabditis elegans* (*C. elegans*) [112,113] or to evaluate locomotive assays in AD *Drosophila melanogaster* model [114]. Different oxidative resistance assays have been performed in vitro with human neuroblastoma SH-SY5Y cells [115,116,117,118], or with cerebellar granule neurons [119]. The Tukey test has also been used to evaluate Tau protein aggregation inhibition assays [119,120].

The Bonferroni method is also used to compare differences between groups using a *t*-test for each pair of groups and is more rigorous than the Tukey test in terms of tolerance of type I errors, this test is used in ANOVA and its multivariate variants and in Pearson correlation analysis. In Bonferroni’s test, the level of significance is modified according to the number of comparisons to be made, so that it can be compensated to make two-to-two comparisons when there are many comparisons, it must be considered that each comparison has a preset α error probability. Azib et al. [61] use the Bonferroni test for post-test analyses in the comparison of mouse behavior, while they use the Tukey test for the comparison of the biochemical parameters evaluated. Hole et al. [121] also used the Bonferroni test to evaluate immunoblotting data in mice treated with epicatechin. In animal models, it has been used to evaluate studies with AChE and BChE and antioxidant capacity [122] and in studies of behavioral difference in treated and untreated animals [123,124]. Studies of measures of antioxidant capacity in human neuroblastoma trials have evaluated the difference between groups treated with dioic oleic extracts and control groups [125].

Among the trials that use pairwise comparison is also Duncan’s method, which has been used to establish differences between pairs of groups with mice that have been induced Alzheimer’s and groups that have or have not been treated with secondary metabolites from Opuntia Ficus [126].

When trials are carried out in which there is a control group, studies that use the Dunnet test are particularly useful, as it is based on a modification of the Student’s *t*-statistic and can find small differences between groups, it also has the disadvantage that it only compares the groups with the control and not among the rest. The Dunnet test has been used for studies in animal models such as *C. elegans* [127,128] and mice [129]. It has also been used in studies with human neuroblastoma cells [62,129] and PC12 cells [130] and to evaluate the aggregation of different Aβ protein fragments [131].

#### 2.4.2. Correlation Analysis, Multivariate Linear Regression and Principal Component Analysis

Bioactivity studies of phenolic compounds provide a wealth of data, and the evaluation of this data is performed in many cases using multivariate analysis. A simple way to establish whether there is a linear relationship between two variables is to use a linear regression model and evaluate the Pearson correlation coefficient for each pair of variables. In this type of statistical analysis, it is assumed that the two variables fit to a normal distribution, that there are no outliers, that the data are representative of the sample, and that a linear relationship between the two variables is expected.

The correlation coefficient can vary between −1 and 1, the sign indicates negative or positive correlation, and the closer the value is to one or minus one the stronger the correlation. On the other hand, it is necessary to evaluate together with the value of the coefficient the *p*-value, which is the probability of observing a non-zero correlation coefficient when the null hypothesis is true; a low *p*-value is consistent with rejecting the null hypothesis that there is no correlation between the data groups. A common value to reject the null hypothesis is a *p*-value of 0.05, if it is lower the correlation coefficient is statistically significant.

Correlation studies have been done to attempt to relate different bioactivities including antioxidant capacity to polyphenolic content, thus Kundo et al. [132] use Pearson’s correlation to relate total polyphenol content, proanthocyanidin content, and antioxidant activity to the cholinesterase inhibitory activity of medicinal herbs *Loranthus globosus* and Guo et al. [66] also evaluated correlations between total polyphenols and total flavonoids and the anti-cholinergic activity, anti-inflammatory activity, and antioxidant activity of thinned peaches extracts. Studies on animal models also use correlation between different sets of data. Valu et al. [64] perform correlation analysis between behavioral and biochemical parameters in zebrafish in which a memory deficit has been induced by scopolamine. A graphical way to see these correlations is heat maps, in which the variables are represented in the rows and columns and in the cells the Pearson correlation coefficient. The color of the cell represents the strength and direction of the correlation, the darker colors indicate greater correlation, Suarez-Montenegro et al. [67] use them to evaluate the neuroprotective potential of the polyphenols present in Tamarillo.

Considering the amount of data generated in most studies, correlation analysis shows only a partial view of the relationships between the different parameters analyzed and polyphenols from different samples, and further studies are needed. After a correlation analysis between different bioactivities of native fruits in Australia, Ali et al. [133] used a biplot representation of the vectors corresponding to the properties evaluated together with the projections of the analyzed fruits and observed that quandong peach had a higher neuroprotective potential due to its anti-cholinergic inhibitory capacity (anti-AChE), although it had a lower content of total polyphenols.

There are different methods to handle large amounts of data without losing relevant information; multidimensional analysis methods make it possible to establish some relationships between different parameters evaluated and the samples being analyzed, and at the same time find correlations between these parameters. PCA is a multivariate technique that is based on the reduction in data taking into account the correlation between them so that the location of the different samples and the parameters analyzed can be represented in a simple way in a reduced coordinate system. PCA is an unsupervised method that allows data sets to be simplified without significantly losing information [134]. Sereia et al. [135] have used PCA to correlate the in vitro activities of plant extracts with their potential neuroprotective action with human neuroblastoma cells. PCA showed that polyphenol fractions extracted in ethyl acetate showed the highest content of phenolic compounds, highest antioxidant capacity, and highest AChE inhibitory activity. Shabbir et al. [136] have also used PCA to evaluate the effects of fermentation on bioactive compounds in black soybeans samples and in germinated black soybeans [137] and their possible action on anti-Alzheimer’s effects.

#### 2.4.3. Experimental Design

Although experimental design is a widely used tool in the optimization of extraction processes whenever several factors may affect the response to be optimized, it has been used less profusely in the extraction of polyphenols for studies in Alzheimer’s disease, and in the studies in which it has been used, the methodology of response surface analysis is used to evaluate parameters related to anti-AChE capacity [67] or in survival studies in in vivo trials [138].

In the case of polyphenol extracts, experimental design has been used to optimize the extraction conditions that allow extracts with greater antioxidant capacity, highest AChE inhibitory activity or any other property with an anti-Alzheimer’s effect to be obtained. The optimization of the extraction of bioactive compounds in Tamarillo [67] was carried out using a central composite design and using the response surface methodology, two factors (solvent composition and temperature) and three levels have been considered, and among the responses to be optimized was the anti-AChE capacity, a response that was adjusted to a quadratic model that allows to observe that temperatures of 180 °C allow extracts to be obtained rich in flavonoids, polyphenols, and carotenoids with promising antioxidant, anti-inflammatory, and anti-AChE activity. A Box–Behnken design and response surface analysis used Yan et al. [138] to optimize the extraction of leaves of *Alsophila spinulosa* and extracts were used to increase the survival of *C. elegans* subjected to high temperature.

## 3. Polyphenols and Their In Vitro Impact on AD Treatment

In the context of AD treatment, polyphenols have been the subject of extensive in vitro research on their potential neuroprotective effects. In nature, polyphenols are synthesized via the shikimate and malonate pathways [139], leading to a mixture of phenolic compounds [140], whose identity and concentration is often unexplored. Therefore, multiple in vitro studies have relied on pure polyphenols, referring to these compounds in their isolated form, devoid of any accompanying substances from the original biogenic source. These are often extracted and purified for research purposes, allowing a more controlled analysis of their properties and effects. Nevertheless, this approach presents certain drawbacks, such as the lack of synergistic effects, as well as the lengthening, complexity and cost of production procedures. Consequently, the utilization of natural phenolic extracts provides a broader and more representative perspective. Moreover, providing these extracts come from agri-food waste, not only a contribution is made to the cost-effectiveness, efficiency and exhaustive study of the combined effects of several plant compounds, but also to the valorization of waste, thereby supporting the circular economy and the development of society [141].

Exploring the interactions between polyphenols and proteins involved in AD is a critical aspect of understanding their potential therapeutic effects, and in vitro assays are essential to evaluate their bioactive properties [142]. Two key proteins involved in AD are Aβ_42_ and tau. Aβ_42_ is a peptide that forms plaques in the brain, a hallmark of AD. Polyphenols has demonstrated to inhibit the formation of these amyloid plaques, suggesting a potential mechanism for their neuroprotective effects [143]. In healthy neurons, tau protein helps to stabilize microtubules. However, in AD, tau becomes hyperphosphorylated, leading to the formation of neurofibrillary tangles (NFTs), another key feature of AD. Some studies have suggested that polyphenols can inhibit the hyperphosphorylation of tau, further supporting their potential role in AD treatment [144].

Another set of proteins that polyphenols interact with are AChE and BChE. These enzymes are involved in the breakdown of acetylcholine (ACh), a neurotransmitter that plays a key role in memory and learning [145]. Inhibition of these enzymes can increase ACh levels, potentially alleviating some of the cognitive symptoms of AD. Some polyphenols have been found to inhibit AChE and BChE, suggesting another potential mechanism for their neuroprotective effects [146].

**Figure 8:**
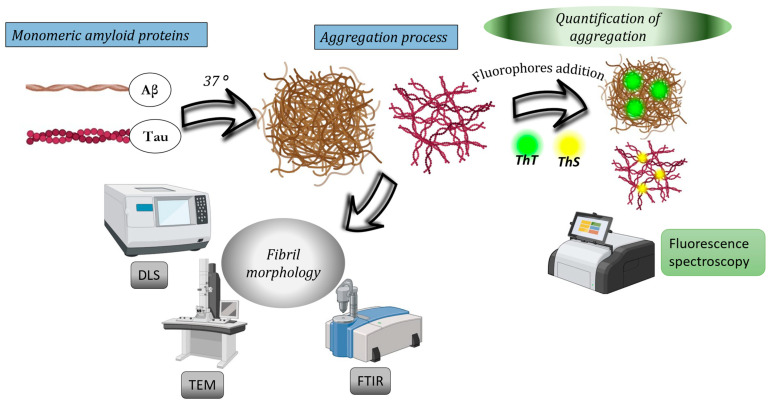
Main bioanalytical methods and techniques employed for the interaction study of polyphenols and key proteins involved in AD pathology.

Integrated approaches using bioanalytical techniques are crucial for studying these interactions. Techniques such as fluorescence spectroscopy, transmission electron microscopy (TEM), dynamic light scattering (DLS), Fourier-transform infrared spectroscopy (FTIR), surface plasmon resonance, and nuclear magnetic resonance can provide valuable information on the binding of polyphenols to these proteins and their downstream effects, allowing elucidation of changes in fibril morphology and aggregation, as well as in cellular morphology [147,148,149] (Figure 8).

On the other hand, primary cell lines derived from neuronal tissue are instrumental in AD research, providing a relevant biological context to study the disease’s pathophysiology and potential treatments. These cell lines include neurons, astrocytes, and microglia, each contributing to the understanding of AD from different angles [150]. Neuronal cell lines, for example, are used to investigate synaptic dysfunction and neurodegeneration, while glial cell lines help elucidate the inflammatory aspects of AD [151].

**Figure 9:**
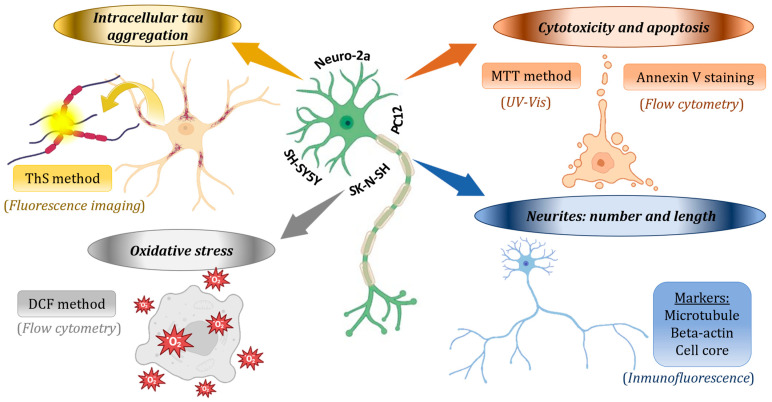
Main bioanalytical methods and techniques for the in vitro neuroprotection assessment of polyphenols using cell lines.

Bioanalytical techniques are pivotal in examining the effects of polyphenols on AD cell lines. Methods such as UV-Vis spectroscopy, immunocytochemistry, flow cytometry, and live-cell imaging allow the observation of the interactions between polyphenols and cellular components, assess cell viability and measure oxidative stress levels [130,152]. These techniques, shown in Figure 9, provide quantitative and qualitative data that contribute to the elucidation of the mechanisms by which polyphenols exert their neuroprotective effects.

Polyphenols have been recognized for their ability to mitigate cytotoxicity in neuronal cells, primarily through their antioxidant properties. They can neutralize free radicals and ROS, reducing oxidative stress, which is a significant contributor to neuronal damage in AD [153]. In vitro studies have shown that polyphenols can also modulate signaling pathways involved in cell survival and apoptosis, further contributing to their neuroprotective potential [154].

This comprehensive section delves into a detailed analytical review that brings to light the current understanding of polyphenols, underscoring their critical role in the context of AD in vitro treatment and research. It offers an analytical deep dive into the most recent findings, emphasizing their pivotal role in advancing in vitro treatment strategies and unlocking new avenues for combating this challenging condition.

### 3.1. Exploring Interactions between Polyphenols and Proteins Involved in AD

The effect of two flavonoids, quercetin and apigenin, on AChE and the Aβ_40_ protein was investigated by Álvarez-Berbel et al. [155]. The study of Aβ_40_ protein aggregation was conducted using fluorescence spectroscopy and thioflavin T (ThT). For this, the excitation was set at 445 nm, and the emission was measured at 480 nm. Incubation of aggregated Aβ protein (20 µM) produced the highest fluorescence intensity, considered as 100%. However, co-incubation with equimolar ratios of apigenin reduced it by 74.8%, while quercetin reduced it up to 85.1%. Thus, it was confirmed that these flavonoids can intercalate between Aβ peptides, forming hydrogen bonds and acting as β-sheet disruptors. The authors justify the high inhibition of quercetin due to the presence of more hydroxyl groups in its structure compared to apigenin. Additionally, the inhibition kinetics were studied for 100 min, measuring factors such as nucleation rate constant, elongation rate, and lag time. Once again, quercetin showed a higher nucleation rate constant (4.5 times higher than Aβ protein alone) than apigenin (3.2 times higher). This is due to interactions between Aβ_40_ and flavonoids in the initial stages of nucleation, leading to aggregates with non-amyloidogenic conformations. Apigenin did not affect the elongation rate, while quercetin reduced it by half. The lag time was reduced in the presence of both flavonoids, being shorter for quercetin. The aggregation profile was different for both flavonoids, as the time to reach half of the total aggregation was shorter in the presence of apigenin than in Aβ protein alone, while it increased in the presence of quercetin. Furthermore, the inhibition of AChE-induced amyloid aggregation (4 µM) was studied, and it was observed that the inhibition was lower than in the absence of AChE, as flavonoids could bind to AChE, leaving fewer free flavonoids to inhibit Aβ_40_. Once again, quercetin showed the most inhibition, reducing absorbance by 50%, while apigenin only reduced it by 35%. The presence of AChE reduced the nucleation rate constant by half and the 50% aggregation time, while increasing the elongation rate constant compared to Aβ protein alone. The morphology of the fibrils was studied using TEM, DLS, and FTIR. In TEM micrographs taken at 120 kV with negative staining using UranyLess, the presence of AChE led to an increase in amyloid aggregation. In the presence of quercetin and apigenin, shorter fibrils and amorphous aggregates were observed. In the absence of AChE, DLS showed that the size of amyloid aggregates decreased by 25% and 50% under the effect of apigenin and quercetin, respectively. In the presence of AChE, only quercetin slightly reduced the size of the aggregates. The secondary structure observed by ATR-FTIR was similar under all conditions, showing two intense peaks at 1611 and 1631 cm^−1^, related to β-sheet structures.

The inhibition of AChE by quercetin and apigenin was studied using the Ellman method and applying UV-Vis spectroscopy. Both flavonoids inhibited AChE activity, with half maximal inhibitory concentration (IC_50_) values of 40.7 and 52.9 µM for quercetin and apigenin, respectively. Once again, due to the higher presence of hydroxyl groups, quercetin offered a better value in terms of neuroprotection. Furthermore, the results of these authors demonstrated that these flavonoids are non-competitive inhibitors, as observed by the reduced values of Vmax and KM in the Lineweaver–Burk plot. Through fluorescence spectroscopy, it was confirmed that both flavonoids produced a quenching effect on AChE fluorescence, indicating binding of quercetin and apigenin to Trp residues in the active sites of the enzyme, with a higher probability of binding to the peripheral site. Additionally, this binding occurred with a 1:1 stoichiometry, with apigenin showing the highest affinity constant.

Andrade et al. [156] investigated the neuroprotective effect of three adducts derived from flavonoid isomers, such as catechin and epicatechin. Specifically, epicatechin-pyrogallol (EPIC-PYR), catechin-pyrogallol (CAT-PYR), and catechin-phloroglucinol (CAT-PhG) were studied for their impact on tau protein aggregation. In the experimental setup, tau protein (50 µM) was aggregated using heparin for 72 h. Subsequently, flavonoid derivatives (5 µg·mL^−1^) were added, and the aggregation was measured using thioflavin S (ThS) (6.25 µM) fluorescence spectroscopy over a period of 10 days. A 50% reduction in tau protein aggregation was observed with CAT-PhG on the fourth day, reaching maximum inhibition (70%) on the eighth day. In contrast, CAT-PYR and EPIC-PYR exhibited a different profile, reaching maximum inhibition (83%) on the fourth day, and decreasing to 70% on the eighth day. This positions catechin and epicatechin-derived adducts as promising molecules for inhibiting tau protein aggregation.

Cannflavin A is a flavonoid present in the *Cannabis sativa* L. plant. Eggers et al. [130] compared its neuroprotective activity against Aβ_42_ and lipid peroxidation with two other flavonoids from the same plant: mimulone and diplacone. First, they evaluated the ability to inhibit the kinetics of Aβ_42_ protein fibrillation (10 µM) at 37 °C for 48 h, measuring fluorescence using the ThT method (10 µM), in the absence and presence of the three flavonoids (100 µM). Measurements were taken every 10 min, with excitation fixed at 446 nm and emission at 490 nm. Mimulone was unable to halt fibrillation, unlike cannflavin and diplacone, which showed a drastic reduction in the kinetics of amyloid fibril formation. Using TEM, after 48 h of incubation at 37 °C and negative staining with 2% uranyl acetate, a significant decrease in the density of aggregates of Aβ_42_ protein (10 µM) was observed in the presence of 100 µM of diplacone, and to a lesser extent with cannflavin A and mimulone. Docking studies revealed the significant ability of cannflavin A to bind to Aβ_42_ oligomers, although all three flavonoids interacted with the hydrophobic groove of the protein.

In the study conducted by Choi et al. [157], the effect of biflavonoids similar to amentoflavone on fibrillation and aggregation of the Aβ_42_ protein was assessed. Specifically, the studied biflavonoids included amentoflavone (four hydroxyl groups), bilobetin (three hydroxyl groups), sequoiaflavone (three hydroxyl groups), sotetsuflavone (three hydroxyl groups), podocarpuflavone (three hydroxyl groups), ginkgetin (two hydroxyl groups), isoginkgetin (two hydroxyl groups), and sciadopitysin (one hydroxyl group). To monitor amyloid aggregation ([Aβ_42_] = 20 µM), fluorescence spectroscopy and ThT (5 µM) were employed. The protein was incubated with amentoflavone (0.08, 0.4, 2, and 10 µM) at 37 °C for 24 h, with the excitation wavelength fixed at 460 nm, and emission measured at 508 nm every 10 min. Maximum aggregation was observed in the Aβ_42_ control alone at 12 h, while it was dose-dependently inhibited in the presence of amentoflavone. The authors noted that the fluorescence emission of the control decreased over time due to photobleaching during successive measurements. The authors calculated the amyloid inhibition IC_50_ values for the eight biflavonoids, with the best results for podocarpuflavone ((0.19 ± 0.01) µM), amentoflavone ((0.26 ± 0.03) µM), and sequoiaflavone ((0.29 ± 0.01) µM), followed by bilobetin ((1.53 ± 0.20) µM), isoginkgetin ((1.80 ± 0.38) µM), and sciadopitysin ((2.28 ± 0.23) µM), and finally ginkgetin ((4.92 ± 1.31) µM) and sotetsuflavone ((8.70 ± 1.21) µM). Therefore, all biflavonoids generally showed promising results, with IC_50_ values below 10 µM. Subsequently, the ability to disaggregate fibrils was studied by exposing preformed fibrils to biflavonoids for 7 h, with emission measured every 2.5 min. In this case, considering the IC_50_ values, amentoflavone exhibited the best results in the disaggregation of preformed fibrils ((0.59 ± 0.19) µM). Most biflavonoids showed values below 10 µM: podocarpuflavone ((1.45 ± 0.40) µM), sequoiaflavone ((2.04 ± 0.79) µM), bilobetin ((2.45 ± 1.28) µM), and ginkgetin ((6.81 ± 4.08) µM), while isoginkgetin, sotetsuflavone, and sciadopitysin had IC_50_ values above 10 µM. AFM was used to evaluate the morphology of amyloid fibrils under the influence of amentoflavone (25 µM). In the absence of amentoflavone, the conversion of monomers into long fibrils was observed, while in the presence of this biflavonoid, amorphous aggregates of Aβ_42_ were observed. SDS-PAGE confirmed that amentoflavone was able to disaggregate amyloid fibrils, forming amorphous aggregates without generating toxic oligomers of this protein. The authors conclude that these biflavonoids, containing hydrophilic and hydrophobic residues, are capable of binding to peptides, oligomers, and Aβ fibrils through hydrogen bonds and both hydrophobic and aromatic interactions, much more effectively than monoflavonoids. The most effective biflavonoid was amentoflavone, as it possessed four hydroxyl groups, compared to others that had only one, two, or three.

As previously mentioned, plant extracts are an effective way to obtain polyphenols naturally. A clear example is the case of rock tea (RT), also known as *Jasonia glutinosa* (L.) de Candolle (DC), whose ethanolic extract is rich in caffeic acid and quercetin, a type of flavonoid. Les, F. et al. [112] employed the Ellman method and observed that this extract could inhibit the activity of AChE. They studied concentrations ranging from 0.63 mg·mL^−1^ to 20 mg·mL^−1^, measuring absorbance at 405 nm every 13 s. The RT extract showed an IC_50_ value of 4.5 mg·mL^−1^, while galantamine used as a reference showed an IC_50_ of 0.1 mg·mL^−1^. Monoamine oxidase A (MAO-A) is known to degrade neurotransmitters such as serotonin, norepinephrine, and dopamine, making its inhibition particularly relevant in the search for an AD treatment. Les et al. observed, using UV-Vis spectroscopy at 490 nm every 5 min for a total time of 30 min, that the RT extract exhibited MAO-A inhibition within the studied range (0.0001–10.0000 mg·mL^−1^). Specifically, the obtained IC_50_ was 76.34 µg·mL^−1^, although clorgyline used as a reference had an IC_50_ of 0.12 µg·mL^−1^. Additionally, they monitored tyrosinase (TYR) inhibition by measuring absorbance at 475 nm, with extract concentrations ranging from 0.001 to 5.000 mg·mL^−1^. They observed 100% enzyme inhibition with 5 mg·mL^−1^ of extract, with an IC_50_ of 1.05 mg·mL^−1^, again lower than the reference compound, kojic acid (IC_50_ = 0.004 mg·mL^−1^). In summary, the ethanolic extract of RT has demonstrated the ability to inhibit enzymes involved in neurotransmission, such as AChE, MAO-A, and TYR, leading the authors to suggest its potential application as a stimulant, memory enhancer, and antidepressant.

*Desmodium elegans* DC., a member of the Fabaceae family, is employed in traditional medicine for various purposes. Its roots act as diuretics, and its leaves are utilized for wound healing. Traditionally, this plant has been used to alleviate fever, asthma, convulsions, and paralysis. Mahnashi et al. [122] characterized the methanolic extract of *D. elegans* using HPLC-DAD, identifying the presence of 16 different types of polyphenols, with the most abundant being GA (239 mg·g^−1^), (−) and (+)-catechin (211.79 mg·g^−1^ and 71.34 mg g^−1^, respectively), kaempferol-7-O-rutinoside (53.67 mg·g^−1^), and *p*-coumaroylhexose (41.2 mg·g^−1^). This methanolic extract was sequentially fractionated with n-hexane, chloroform, and ethyl acetate, yielding four different types of extracts. Using the DTNB method and measuring absorbance at 412 nm with UV-Vis spectroscopy, the inhibitory ability of these extracts against AChE and BChE was studied. The extracts with the highest inhibitory capacity against AChE were methanolic and chloroform, with inhibition of 89.00% and 86.50%, respectively, at a concentration of 1 mg·mL^−1^. The calculation of IC_50_ confirmed the superior results of chloroform (47.32 µg·mL^−1^) and methanolic extract (62.34 µg·mL^−1^), compared to the inhibitions of ethyl acetate (180.91 µg·mL^−1^) and n-hexane (205.21 µg·mL^−1^) extracts. Additionally, galantamine used as a reference offered an IC_50_ = 4.74 µg·mL^−1^. The methanolic, chloroform, and n-hexane extracts showed the highest inhibition of BChE, being 91.12%, 84.36%, and 83.57% at extract concentrations of 1 mg·mL^−1^. Regarding IC_50_ values, the chloroform extract was the most effective (45.98 µg·mL^−1^), followed closely by methanolic (58.87 µg·mL^−1^), ethyl acetate (192.56 µg·mL^−1^), and hexane (210.14 µg·mL^−1^) extracts. Galantamine exhibited an IC_50_ = 2.32 µg·mL^−1^. These results underscore the potential application of *D. elegans* extracts in alleviating the neurotoxic effect caused by AChE and BChE enzymes in AD.

One of the main ingredients in beer is hop, also known as *Humulus lupulus* L., and its consumption is associated with improved cognitive functions and attention. To investigate its bioactive properties, Palmioli et al. [128] employed four different hop varieties, such as Cascade (HC), Saaz (HS), Tettnang (HT), and Summit (HSu), obtaining hydroalcoholic extracts with 10% EtOH and characterizing them by UPLC-PDA-HR-MS. A total of 42 compounds were identified, mainly from the family of CGAs, proanthocyanins, and glycosyl flavonoids. Specifically, the presence of caffeoyl, feruloyl, and p-coumaroylquinic acid derivatives, and various glycosyl-flavonols, such as rutin, astragalin, and spiraeoside, was observed. Quercetin and kaempferol were the major aglycones. These hop extracts were found to be rich in monomers and oligomers of flavan-3-ols, such as catechin and procyanidin. The total polyphenol content, obtained using the Folin method, was (106.2 ± 0.7) mg·GAE g^−1^ for HSu, (93 ± 1) mg·GAE g^−1^ for HT, (71 ± 3) mg·GAE g^−1^ for HS, and (64.5 ± 0.8) mg·GAE g^−1^ for HC. The aggregation of the Aβ_42_ protein was studied using the ThT method employing fluorescence spectroscopy. For this, 2.5 μM of Aβ_42_ was incubated for 24 h at 37 °C in the presence of 20 μM ThT and presence or absence of each extract at a concentration of 0.25 mg·mL^−1^. All extracts showed a fairly similar inhibition of Aβ protein aggregation, with HT being the most effective (99% inhibition) and HC being the least effective (85% inhibition). Atomic force microscopy (AFM) confirmed the anti-amyloidogenic properties of the four hop extracts. While all of them were effective in this aspect, as there were almost no protein aggregates, and when present, they were amorphous and HT emerged once again as the most promising, as no aggregates were observed. In samples incubated with HS, small aggregates ranging in size from 5–10 nm were found, while with HSu and HC, some clusters without a defined structure of between 20 and 100 nm were found. It is particularly relevant to highlight that in the presence of the four hop extracts, neither fibrils nor protofibrils of the Aβ protein were observed, eliminating the possibility of amyloid plaques formation, and opening the door to their application with neuroblastoma cells.

### 3.2. Neuroprotective Effects in Cell Lines: Mechanisms and Effectiveness

Andrade et al. [156] conducted in vitro studies on flavonoid isomer-derived adducts, including catechin and epicatechin, namely EPIC-PYR, CAT-PYR, and CAT-PhG, using the Neuro-2a cell line. Employing the 3-(4,5-dimethylthiazol-2-yl)-2,5-diphenyltetrazolium bromide (MTT) method and UV-Vis spectroscopy, it was observed that none of the flavonoid derivatives exhibited cytotoxicity within the concentration range of 0.5 g·mL^−1^ to 100.0 g·mL^−1^. Immunofluorescence, along with alpha-tubulin, a microtubule-associated protein marker, phalloidin, a β-actin protein marker, and Topro, a cell core marker, was utilized to examine the effect of flavonoids on cell morphology. In the concentration range of 0.5–5.0 µg·mL^−1^, all derivatives increased the number and length of neurites while reorganizing the neuron cytoskeleton. Among the derivatives studied, EPIC-PYR exhibited the most significant effects, rendering it the most promising. Therefore, a neurogenic effect promoted by all three flavonoid derivatives was observed, contributing to the reversal of neurodegeneration and cell death induced by AD.

Another relevant polyphenol in this context is epigallocatechin gallate (EGCG), a flavonoid found in high amounts in green tea [158]. In this study, its neuroprotection was investigated using the SH-SY5Y cell line and the MTT method. At 24 h, under the individual effect of Pb (5 µM), viability was reduced to 50.3%, while with Aβ_40_ (60 µM) it was 42.8%, and with Aβ_25–35_ (8 µM) it decreased to 46.9%. Double combinations produced a synergistic effect, with viability of 18.9% for Pb + Aβ_40_, 28.2% for Pb + Aβ_25–35_, and 21.2% for Aβ_40_ + Aβ_25–35_. Finally, the combination of all three resulted in cytotoxicity up to 94.4%. Co-incubation with EGCG protected against the cytotoxicity of all three species together (53.5% viable cells) and the double combinations: Pb + Aβ_40_ (53.15%), Pb + Aβ_25–35_ (44.1%), and Aβ_40_+ Aβ_25–35_ (41.2%). Using flow cytometry and annexin V staining, apoptosis of cells under the effect of Pb and Aβs for 24 h could be quantified. The annexin V percentage increased compared to the control in the presence of Pb (32%), Aβ_40_ (28%), Aβ_25–35_ (24.3%), Pb + Aβ_40_ (19.29%), Pb + Aβ_25–35_ (35.68%), Aβ_40_ + Aβ_25–35_ (37.9%), and in the triple combination (43.5%). Under EGCG treatment, cell apoptosis was inhibited compared to the control by 22%, 10%, 11%, 6.2%, 4.1%, 3.8%, and 5.1%, respectively. Caspase-3 levels increased by 2950% under the effect of the three neurotoxic species, 1109–2006% with double combinations, and 764–1500% in the presence of individual species. However, co-incubation with EGCG reduced the percentage to 188% in the triple combination, 246–730% in double combinations, and 246–371.1% in the presence of individual species, demonstrating the efficacy of this flavonoid in reducing apoptosis. Flow cytometry revealed an increase in the percentage of damaged DNA and inhibition of cell proliferation by Pb, Aβ_40_, Aβ_25–35_, double combinations, and the triple combination ranging from 0.2% to 68.9%. EGCG protected against both neurotoxic effects, reducing them between 0.33% and 35.43%. Levels of ROS determined by fluorescence with dichlorofluorescein (DCF) increased in the presence of Pb and Aβs by 13.65–62.1%, while treatment with EGCG reduced ROS levels by 2.41–7.7% compared to the control. Collectively, the findings derived from the current investigation indicate the harmful consequences brought about by the concurrent presence of Pb and Aβ, particularly in relation to cell viability, proliferation, and oxidative stress. Consequently, this combination results in apoptosis, a process that can be alleviated through exposure to EGCG.

Eggers et al. [130] evaluated the cytotoxicity of cannflavin A, as well as mimulone and diplacone, through MTT method on the semi-differentiated PC12 cell line. Diplacone was the most toxic flavonoid, with a concentration of 1 µM selected due to its low cytotoxicity for subsequent experiments. For mimulone and cannflavin A, a concentration of 10 µM was chosen due to their negligible toxicity. Tert-butyl hydroperoxide (t-php) was used as an inducer of lipid peroxidation, causing a devastating effect on cells at concentrations between 100 and 400 µM. While flavonoids could not protect against lipid peroxidation in that concentration range, cannflavin A did so at low concentrations of t-php (0–50 µM). When cytotoxicity was induced with Aβ_42_ (0–1 µM) during 48 h of incubation, cell viability was reduced to 60%. The use of cannflavin A (10 µM) significantly inhibited the neurotoxicity produced by the amyloid protein at all concentrations studied. On the other hand, diplacone (1 µM) and mimulone (10 µM) showed partial inhibition of this neurotoxicity, with slightly positive effects but lower than cannflavin A. Fluorescent imaging was used to visualize the aggregation of Aβ_42_ and the effect of cannflavin A on the morphology of PC12 cells. Cells were fixed and stained with Amylo-glo (Aβ-binding fluorophore), acridine orange (nuclei and cytoplasm fluorophore), and phalloidin-TRITC (cytoskeleton staining). In the presence of Aβ_42_, PC12 cells progressively lost neuritic projections and filopodia. However, in the presence of cannflavin A, amyloid aggregates partially decreased. These results indicate the high neuroprotective potential of cannflavin A in particular, and to a lesser extent, the other two flavonoids evaluated in this study: mimulone and diplacone.

Martínez-Díaz et al. [159] investigated the effect of resveratrol, a stilbene, on the C6 cell line. Initially, cell viability at 24 and 48 h was assessed using the MTT method and UV-Vis spectroscopy. Concentrations of 12.5 and 25 µM of resveratrol showed no cytotoxicity compared to the control, while 50 and 100 µM resulted in a considerable reduction in viability. Therefore, the authors selected the concentration of 25 µM to study its effect on tau protein and its hyperphosphorylation using ELISA and Western blot methods. Resveratrol reduced the expression of tau protein by 33%, along with decreasing its hyperphosphorylation at the Ser_262_ residue. Furthermore, there was a 23% reduction in the expression of β-secretase, responsible for the amyloidogenic processing of APP. This study demonstrated the in vitro capacity of resveratrol to combat tau neuropathology, as well as reduce the amyloidogenic pathway of APP.

Chen et al. [160] investigated the effects of punicalagin (PU), a type of tannin, on BV2 and Neuro-2a cell lines. The protective effect against lipopolysaccharide (LPS)-induced cytotoxicity in BV2 microglial cells was measured using the MTT method. On its own, PU did not cause a reduction in viability at 1, 5, or 10 µg·mL^−1^, and it was statistically similar to the control. However, while LPS (100 ng·mL^−1^) induced 50% cytotoxicity, co-treatment with PU reduced cytotoxicity to 70%, 80%, and 90% for PU doses of 1, 5, and 10 µg·mL^−1^, respectively. Furthermore, the ELISA assay showed that PU was able to reduce LPS-induced neuroinflammation, lowering the expression levels of pro-inflammatory cytokines such as interleukins (IL-18, IL-1β, and IL-6) and tumor necrosis factor-alpha (TNF-α), induced by LPS. This anti-inflammatory activity was also evaluated in the Neuro-2a neuronal cell line, demonstrating that PU had a protective effect on neurons. Additionally, LPS increased malondialdehyde (MDA) levels and the activity of enzymes such as superoxide dismutase and glutathione peroxidase, with these levels decreasing in a dose-dependent manner under co-treatment with PU. Treatment of Neuro-2a with LPS decreased synaptic density, lowering levels of the presynaptic protein synaptophysin and the postsynaptic protein PSD-95. However, application of the tannin PU successfully reversed the reduction in these synaptic proteins to levels similar to the control at a concentration of 10 µg·mL^−1^. Together, these results suggest that PU holds promise as a potential safeguard for cognitive function in the treatment of age-associated neurodegenerative diseases. However, the authors suggest that it is advisable to conduct comparative studies with other anti-inflammatory and anti-aging agents, such as resveratrol, to clarify whether the anti-aging effects observed with PU result primarily from its antioxidative activity or other mechanisms. Additionally, they stated that none of the PU preparations have undergone medical clinical trials thus far. Consequently, further clinical studies are imperative to formulate therapeutic strategies incorporating PU.

Tannic acid (TA) is a tannin composed of ten GA molecules. While GA can cross the BBB, TA cannot penetrate the BBB due to its large size. For this reason, Hu et al. successfully encapsulated TA in liposomes, considering it as a vehicle to reach the central nervous system (CNS). Initially, the cytotoxicity of both empty liposomes and TA-loaded liposomes was evaluated in mouse brain microvascular endothelial cell (bEnd.3) lines, used to study BBB permeability, and human neuroblastoma cells (SK-N-SH), employed for tau protein aggregation studies. Through incubation of liposomes and TA-loaded liposomes with bEnd.3 cells (0–25 µM) for 24 h, it was observed that neither affected cell viability. However, in SK-N-SH cells (0–3 µM), liposomes increased cell proliferation, validating the choice of this vehicle. Nonetheless, in the presence of TA-loaded liposomes, SK-N-SH cell viability decreased (to 80% at 3 µM), prompting the investigation of TA cytotoxicity alone, which showed 75% viability at 3 µM. Subsequently, the neurotoxicity of the R3 peptide of the tau protein in SK-N-SH cells was evaluated, resulting in 75% cell viability at 3 µM. Using fluorescence imaging, it was observed that TA-loaded liposomes (labeled with coumarin-6) entered bEnd.3 cells (nuclei stained with SYBR green 1) via endocytosis within 4 h without reducing cell viability, confirming their ability to penetrate the BBB. To assess the effect of the tau protein, R3 peptides (1.5 µM) were used to induce tau aggregation in SK-N-SH cells. This concentration was chosen because it maintained 95% cell viability, thus capable of inducing tau protein aggregation without drastically reducing the cell population. Immunofluorescence imaging revealed signs of tau protein aggregation in SK-N-SH cells after exposure to the R3 peptide for 24 h. Cell nuclei were stained with DAPI (blue fluorescence), and tau protein fibrils with ThS (green fluorescence). Incubation with GA or GA-loaded liposomes partially reduced ThS intensity but did not completely reverse it. Additionally, an increase in DAPI fluorescence was observed, indicating neurogenesis of SK-N-SH cells by GA. Conversely, incubation with TA-loaded liposomes completely reduced ThS intensity, indicative of the elimination of tau protein fibril aggregation in these cells. In conclusion, it has been observed that TA, a polymeric polyphenol composed of 10 GA molecules, can cross the BBB when encapsulated in a liposome. Once inside SK-N-SH cells, TA demonstrated high efficacy in inhibiting tau protein aggregation, reverting its fibrils to an oligomeric state.

Continuing with the application of plant polyphenolic extracts against neurodegenerative diseases, Mattioli et al. investigated *Arabidopsis thaliana*, from the Brassicaceae family, regarding its anti-inflammatory response triggered by Aβ. The ethyl acetate (EtOAc) extract obtained from the raw juice of *Arabidopsis thaliana* was analyzed using UPLC-DAD-MS and UHPLC-DAD-HR-MS/MS, revealing a profile of flavonoids and other polyphenols. Among them, synapic acid stood out as the major polyphenol, along with kaempferol-3-O-glucoside-7-O-rhamnoside, quercetin-di-rhamnoside, and kaempferol-3,7-di-O-rhamnoside. Other polyphenols present in smaller proportions included caffeic acid, quercetin-rhamnoside-hexoside, isorhamnetin-hexoside-rhamnoside, two isomers of di-O-sinapoyl-β-glucose, and luteolin. Although not individually quantified, the authors reported an enrichment of TFC in the EtOAc extract, while anthocyanins and other polyphenols remained in the aqueous phase. While the raw juice contained (0.32 ± 0.02) mg GAE mL^−1^ of total polyphenol content, the EtOAc extract had (0.10 ± 0.01) mg GAE mL^−1^. Furthermore, the raw juice contained (0.88 ± 0.05) mg CGE mL^−1^ of anthocyanins, whose presence was not observed in the extract. In vitro assays were conducted using BV2 murine microglia cells. The first step involved evaluating the extract’s cytotoxicity, revealing no loss of cell viability up to concentrations of 20 µg·mL^−1^, thus this concentration was used in subsequent studies. Moreover, while 25 µM of Aβ_25–35_ reduced cell viability by 35% at 24 h, co-incubation of Aβ_25–35_ and the EtOAc extract partially reversed this cytotoxic effect, achieving 80% cell viability at 24 h post-exposure. To assess the anti-inflammatory effect, cells were treated with Aβ_25–35_ (25 µM) in the presence and absence of the EtOAc extract at 20 µg·mL^−1^. The authors found that pro-inflammatory cytokines (IL-4, IL-6, IL-10, IL-13, IL-1β, TNF-α) increased in the presence of Aβ_25–35_, reaching double the concentration compared to Aβ_25–35_ alone. Treatment with the EtOAc extract did not induce any effect in the first two hours, with cytokine levels statistically equivalent to those of Aβ_25–35_ alone. However, after 24 h of treatment with the extract, levels returned to those of the control, demonstrating the anti-inflammatory effectiveness of the EtOAc extract from *Arabidopsis thaliana*, which began to be observed at 6 h post-application. These findings suggest that this flavonoid-rich extract may help combat AD by reducing both cytotoxicity and neuroinflammation induced in vitro by Aβ.

Palmioli et al. [128] investigated the effect of four extracts from different hop varieties (HT, HS, HSu, and HC) on the SH-SY5Y cell line. Cells were treated with 10 μM Aβ_42_ for 24 h, showing 50% cell viability under those conditions. However, co-incubation with the four extracts partially or completely protected against this cytotoxicity in a dose-dependent manner. When the dose used was 0.5 mg·mL^−1^, it was observed that the most effective extracts were HSu, HT, and HS, completely reversing cytotoxicity, while HC offered the least protection (65% cell viability). The best antioxidant capacity via the DPPH and ABTS methods was observed for the HT extract, so its in vitro antioxidant capacity was evaluated with SH-SY5Y cells. While cell viability decreased to 60% in the presence of 100 μM H_2_O_2_ at 24 h, no cytotoxicity was observed with incubation of the HT extract at 0.1 or 0.25 mg·mL^−1^. As demonstrated, pre-treatment of cells for 1 h with both concentrations of the HT extract partially reversed the cytotoxicity induced by oxidative stress generated by H_2_O_2_, achieving cell viability of 90% and 95% for concentrations of 0.1 and 0.25 mg·mL^−1^, respectively. Other experiments also demonstrated the ability of this hop extract to promote the activation of Aβ autophagy. These combined results of in vitro reduction in oxidative stress and promotion of Aβ catabolism, inducing autophagic pathways of this protein, lead the authors to consider its use as a nutraceutical for AD prevention.

Table 3 reports different phenolic compounds, their source and their in vitro neuroprotective effect on AD.

## 4. Polyphenols and Their Role in AD Treatment: In Vivo Studies

### 4.1. Overview of Key In Vivo Models in AD Research

To evaluate the possible effects of polyphenolic extracts and polyphenols in particular, various animal models have been used, which are crucial for the evaluation of compounds with possible activity against AD. Experiments with rodents play a fundamental role in trying to explain the mechanisms involved in the development and progression of the disease. Rodent models include genetically modified mice [163,164], elderly mice [123,129], and mice that have been induced with AD [61,122,126,160,165]. Table 4 summarizes the main polyphenols used in in vivo animal models against AD. Other animal models have also been used, including *C. elegans* a genetically modified nematode [112,128,166] whose complete genetic sequencing data are known, the fly *Drosophila melanogaster* which is genetically modified in such a way that it shows a strong decrease in neurons in models in which the proteins Aβ_42_ or Tau are overexpressed [162,167,168] and that it is one of the first animals to have a complete genome. Taking into account the occurrence of synaptic and cognitive deficiencies in the disease, many of the studies have been developed considering memory and learning abilities in both mice [61,122,123,126,129,160,164,165] and zebrafish that have been induced with scopolamine disease [64]. Within transgenic mice, the doubly transgenic APP/PS1 has been used [164], this particular model has 30 mutations in the transmembrane glycoprotein type I and 200 mutations in PS1 that are typical of families that develop the disease in early periods [169]. Transgenic models such as female Tg2576 mice have also been used to assess sex-associated cognitive impairment [163], since impairment of cognitive function appears to be sex-dependent [170]. Some studies indicate that there may be a distortion of the results obtained with transgenic mice, given that the pathogenesis and overexpression of some of these genes can produce neuronal effects that are not necessarily related to the pathology of AD. The pathology of AD can be induced in a variety of ways as indicated in Table 4, including treatments with aluminum chloride [61,126], D-galactose [160], scopolamine [122], or injections of Aβ oligomers [165]. In all cases where symptoms of the disease are induced, the animals are classified into different groups so that a statistical assessment using ANOVA can be made, as described in the “2.4 Application of chemometrics in polyphenol characterization“ section, of the results between control animals, which have not been treated, animals that have had symptoms of the disease induced and those in the last group that have been treated have induced symptoms and have also been treated with a polyphenolic extract or a specific polyphenol.

### 4.2. Behavioral Assessments with Models of Alzheimer’s Disease

In AD, changes are observed in the hippocampus and this area of the brain is intimately related to spatial and verbal memory. Considering the distances between species, changes in the hippocampus in rodents are associated with spatial navigation and olfactory behavior [170]. The Morris Water Maze is frequently used to assess long-term spatial learning and memory [61,122,129,160,164]. In this type of experiment, the mice are trained for a variable period, usually five days, in a circular pool containing an escape platform placed 1 cm below the surface of the water. In the first days of training the mice are given a maximum time to locate the platform and stay on it, once the training period is over, the platform is removed and the mice are allowed to swim freely and the number of times they pass through the area in which the platform was is recorded and it is statistically compared between the mice subjected to treatment with polyphenols and the untreated. Improvements in spatial cognitive behavior have been observed in mice treated with extracts of P. lentiscus L. leaves [61], crude extracts of *Desmodium elegans* [122], Sugarcane (*Saccharum officinarum* L.) top extract [129], PU extract [160], and SeNPs coated with dihydromyricetin [164]. The Passive Avoidance test is a test used to assess learning and memory using an adverse stimulus (such as an electric shock) so that test subjects tend to avoid an environment that produces fear in them. A camera with an illuminated compartment and a dark compartment is usually used and in the training stage the animals can explore both compartments, in the following days they receive a slight electric shock in one of the compartments and from then on they will try to avoid the compartment in which they received the shock. This test has been used with mice that have been induced AD with Aluminum chloride and after treatment with Opuntia ficus-indica cladode, peel and fruit pulp extracts have been observed an improvement in the number of times they avoid the chamber in which they will suffer shocks compared to the group of mice that have not been treated [126]. In the case of female models presenting with age-related symptoms of the disease, treatment with olive secoiridoid polyphenols extract also improved the latency to cross through the gate between compartments [123]. Another behavioral test that measures rodents’ willingness to explore new environments is the Y maze test, rodents prefer to investigate a new arm of the maze rather than return to one they have already visited. The test takes place in a Y-shaped maze with three white and opaque arms with an angle of 120° to each other, the mouse is placed in the center of the maze and the number of times that the mice tend to enter the arm that they have not visited are recorded, this test has been used to observe cognitive improvements in mice that had been induced AD with scopolamine and had also been treated with crude extracts of *Desmodium elegans* [122], in models of elderly female mice treated with olive secoiridoid polyphenols extract [123], and in mouse models that have been induced AD with D-gal and treated with PU extract [160]. Other tests such as novel object recognition (NOR) [165] or open field tests [160] have also been used. Some of the behavioral tests used for mice have also been adapted for zebrafish that have been induced to suffer Alzheimer’s symptoms with scopolamine [64].

In the case of behavioral studies with the nematode *C. elegans*, various strategies have been used. Since Aβ-induced toxicity can induce paralysis in nematodes, a paralysis assay is used to track the effect of the protein. The CL4176 strain was treated with different amounts of *Jasonia glutinosa* (L.) DC extract or rock tea [112] at 16 °C for 38 h and the temperature was changed to 25 °C to induce the expression of the Aβ protein and the paralysis to occur. The time for 50% of nematodes to become paralyzed (half maximal paralyzed concentration, PT_50_) increased considerably in the groups treated with the rock tea extract compared to the nematodes that had not undergone any treatment. Similar paralysis studies have been performed with ethyl caffeate [166] and in this case nematodes treated with concentrations between 300 and 600 μM of ethyl caffeate showed an increase in mobility of up to 133.9% when the temperature is changed to 25 °C compared to the motility of untreated CL4176 strain nematodes. The protective effect of aqueous hop extractors on the transgenic variety CL2006 in which the paralysis phenotype is caused by the deposition of Aβ_3-42_ fibrils in muscle cells has also been studied, a dose of 12.37 μg·mL^−1^ of hop tea (*Humulus lupulus* L.) is calculated as IC_50_ value (43).

Regarding *Drosophila melanogaster* flies, the Capillary Feeder assay has been used to measure feed intake in flies and their chances of survival, and mobility is measured as ascent capacity by sterile vials [114]. The life span of flies can increase up to 66.1% when treated with p-coumaric acid, mobility studies carried out by genus in different species show a protective effect of p-coumaric acid against the symptoms of AD and that in turn depends on the sex of the flies. Mobility tests have also been conducted on *Drosophila melanogaster* flies treated and untreated with a polyphenolic extract of *Arabidopsis thaliana* and flies treated with the extract have been observed to increase their climbing ability [162].

### 4.3. Histopathological Examinations: Polyphenols’ Effects in the Brain

Histopathological studies have been performed in the brains of mice and in the case of the SAMP8 model, an immunohistochemical study of the cerebral cortex has been performed and the levels of monoamines (dopamine, norepinephrine and serotonin) in the tissue have been evaluated by sandwich ELISA, and an ELISA assay has also been used to determine ACh levels [129]. RNA extracted from the brain was also evaluated and analyzed once amplified and biotined; oral administration of Sugarcane Top Ethanolic Extract restored neurotransmitter levels in the cerebral cortex of SAMP8 mice. On the other hand, microarrays analysis of the cerebral cortex allows us to investigate the underlying mechanisms when Sugarcane Top Ethanolic Extract is administered; 689 dysregulated genes have been found, 339 have increased, and 350 have decreased if comparing mice that have undergone treatment with the extract against control mice. Of these, 13 genes are associated with neuronal protection, which could explain the reversal of spatial memory deficits in SAMP8 mice treated with the ethanolic extract of Sugarcane top. In the case of mice that have been induced with Alzheimer’s symptoms by aluminum chloride [126], the levels of catecholamines (norepinephrine, dopamine, serotonin), AChE, and markers of oxidative stress, lipid peroxides, and mediators of inflammatory processes (NF-kβ, IL-10 and TNF-α) were also studied. Mice treated with Opuntia ficus-indica extracts showed higher levels of norepinephrine, dopamine, and serotonin than those treated with aluminum chloride alone, the latter also showed lower levels of interleukin-10 (IL-10) and elevated levels of nuclear factor kappa (NF-kβ). in the control group and decreased in the group of animals treated with Opuntia ficus-indica extract. All animals treated with the extract showed a significant decrease in brain levels of NF-kβ and TNF-α. To relate the effects of the extract on the brain, a Docking study was conducted that suggests that the extract components of Opuntia ficus-indica may serve as inhibitors of AChE and serotonin transporters.

In the case of male C57BL/6J mice that have been induced with D-gal and have also been treated with PU [160], studies have been conducted of possible damage to the brain, liver, spleen, kidneys and heart, and fewer lesions of these organs have been observed when the mice have been treated with PU. To evaluate the possible damage to the brain and its possible improvement with treatment with PU, hematolysin eosin, and Nissl staining assays have been carried out, which showed that the older group of mice had aberrations in the hippocampus that were observed to a lesser extent in the mice treated with PU. The Nissl trial showed that the number of surviving neurons is lower in the group of aged mice treated with D-gal, while an increase in normal neurons is seen in the aged mice treated with PU. Studies conducted on female Tg2576 mice with rosmarinic acid [163] indicate that treatment with this polyphenol is able to suppress the accumulation of Aβ, on the other hand, DNA analysis using microarrays showed that an improvement of the dopamine signaling pathway occurs when mice are given rosmarinic acid. Analysis of monoamines in the cerebral cortex showed that they increase when mice are fed rosmarinic acid, which may be beneficial against AD symptoms.

### 4.4. Bioanalytical Assays: Polyphenols’ Influence in AD Models

The theory of aging attributed to oxidative stress suggests that declines in physiological functions with age result from the gradual buildup of damage caused by ROS. Advanced glycation end products have been identified as a hallmark of aging, contributing to heightened oxidative stress and inflammation. Consequently, safeguarding immunity and preserving redox balance are crucial for thwarting and slowing down the aging process in organisms.

Lately, there has been a surge of interest in glial cells, notably astrocytes and microglia. A wealth of evidence indicates that gliosis is intricately linked to brain pathology and the decline in memory associated with premature aging, as well as in aging animal models exposed to D-galactose. Hence, it is imperative to contemplate the comprehensive targeting of neuroinflammation concerning various diseases, as it may represent the most efficient and effective approach to safeguarding against neuronal damage in aging and AD mice.

The NOD-like receptor protein 3 (NLRP3) inflammasome, a pattern recognition receptor involved in signal transduction, is primarily found in immune cells like microglia. Activation of NLRP3 can induce neuroinflammation by stimulating the production of proinflammatory cytokines such as IL-1β and IL-18. These cytokines, upon release, can further activate additional inflammatory cells to release more inflammatory mediators, thus amplifying various downstream damage signaling pathways. This sets off an inflammatory cascade, ultimately resulting in neuronal cell damage during the aging process [160].

Chen et al. evaluated the effect of PU, [2,3-(S)-hexahydroxydiphenoyl-4,6-(S,S)-gallagyl-D-glucose, a major polyphenol component of *Punica granatum* with potent antioxidant and anti-inflammatory properties, on aging mice. The study examined the protective impact of PU on Neuro-2a cell damage induced by BV2 microglia-triggered neuroinflammation. Results revealed that PU effectively mitigated deficits in learning and memory and thwarted neuroinflammation, as evidenced by reduced microglial activation and astrocytosis. Moreover, its supplementation led to decreased MDA and ROS species levels while also inhibiting the activation of the NLRP3 inflammasome, resulting in lowered levels of inflammatory cytokines in both accelerated aging and naturally senescent mouse models [160].

On the other hand, Les et al. evaluated polyphenol-rich extracts from *Jasonia glutinosa* (L.) DC, an herbal tea plant used in traditional medicine, and in C. *elegans* as an in vivo Alzheimer’s model. Ethanolic extracts reduced juglone-induced oxidative stress, increased the lifespan, and prevented paralysis in worms [112].

Mattioli et al. also demonstrated the anti-inflammatory activity of polyphenolic extracts from *Arabidopsis thaliana* in in vivo models. They evaluated the effects of ethyl acetate extracts on the inflammatory response in *Drosophila melanogaster* flies expressing the human Aβ_1–42_ at the glial level, confirming that plant extracts significantly restored the locomotor activity of these flies, thus exhibiting neuroprotective properties [162].

Using a similar AD in vivo model, Hui Ping Tan et al. evaluated the effects of *p*-coumaric acid, confirming that this polyphenol partially reversed the rough eye phenotype, notably extending the lifespan of AD *Drosophila* and improving mobility in the majority of AD *Drosophila* in a sex-specific manner. The feeding of *p*-coumaric acid can reduce inflammatory reactions and cellular senescence, potentially decreasing the amount of Aβ_42_ [114].

Neuroinflammation processes in AD have also been studied in mice. Tomaselli et al. synthesized complex polyphenols derived from natural sources and assessed their capacity to inhibit the formation of Aβ_42_ oligomers. These oligomers are known to be the most detrimental species, responsible for synaptic dysfunction, neuroinflammation, and neuronal demise, which ultimately drive the onset and advancement of AD. In vivo studies conducted on a mouse model induced with Aβ_42_ oligomers demonstrated that the most effective polyphenolic derivative (PP04) mitigated the adverse impacts of Aβ_42_ oligomers on memory and the activation of glial cells [165].

Yang et al. have also evaluated in mice the effect of selenium nanoparticles coated with dihydromyricetin, a natural polyphenol, and decorated with peptide Tg (TGNYKALHPHNG), on neuroinflammation. Synthetized nanoparticles successfully inhibited Aβ aggregation and reduced inflammatory cytokine secretion via the NF-κB pathway in the brain of APP/PS1 mice, also repairing the gut barrier and regulating the population of inflammatory-related gut microbiota such as *Bifidobacterium*, *Dubosiella*, and *Desulfovibrio* [164].

## 5. Emerging Trends and Conclusions

The exploration of polyphenols as potential therapeutic agents in AD has opened up new avenues in neurodegenerative research. The multifaceted nature of polyphenols, encompassing their antioxidant properties, modulation of signaling pathways, and interactions with gut microbiota, underscores their potential as neuroprotective agents.

Emerging trends in the field point towards the increasing application of the circular economy concept to the extraction of polyphenolic compounds. This approach not only aligns with principles of sustainability and environmental responsibility but also allows for the utilization of plant-based waste materials as valuable sources of polyphenols. Yet the lack of consistent phenolic composition characterization hinders comparative analysis. Overall, the flavonoid group stands out as the most extensively researched polyphenol family for neuroprotection against AD. Notable flavonoids like quercetin, apigenin, catechin, epicatechin, epigallocatechin gallate, cannflavin A, amentoflavone, mimulone, and diplacone are often the focus of neuroprotection studies. Additionally, other polyphenols such as stilbenes (notably resveratrol), tannins (like punicalagin), and phenolic acids (especially gallic acid) are recognized for their neuroprotective properties.

In vitro studies have been instrumental in elucidating the interactions between polyphenols and key proteins involved in AD, such as Aβ_42_ and tau. These studies have demonstrated the potential of polyphenols to inhibit the formation of amyloid plaques and neurofibrillary tangles, hallmarks of AD. Furthermore, polyphenols have shown promise in mitigating cytotoxicity in neuronal cells, primarily through their antioxidant properties and their ability to modulate cell survival and apoptosis signaling pathways.

Complementing these in vitro findings, in vivo studies using various animal models have provided further evidence of the neuroprotective potential of polyphenols. These studies have been carried out in many cases with elderly mice, which allows us to make better approximations about cognitive impairment, and with female mice, which can offer very valuable information to explain the higher incidence of the disease in women. In conclusion, in vivo results have demonstrated that certain polyphenols can cross the BBB, decrease amyloid levels, reduce plaque formation, and modulate neuroinflammation and oxidative stress. Considering the criteria for database selection, a total of 122 studies have been carried out on AD utilizing polyphenols. Among these, 56 were exclusively in vitro, 51 solely in vivo, and 15 combined both in vitro and in vivo approaches. In cases where complex phenolic extracts are employed, their precise identification and quantification often remain unaddressed. This highlights the necessity for concurrent in vitro and in vivo assays, not only for the isolated standard polyphenol but also when used in conjunction, particularly with phenolic extracts, to garner more comprehensive insights into their potential neuroprotective impact.

Despite these promising findings, several challenges remain. One of them is the imperative to analyze the extracts used in these studies in terms of individual polyphenols to correlate their phenolic composition with the effects they produce. Many studies characterize these extracts in terms of the total amount of polyphenols, which is insufficient for a comprehensive understanding of their neuroprotective effects. To achieve this individual characterization, it is necessary to resort to chromatographic techniques, primarily HPLC, coupled to mass analyzers (HPLC-MS) for confirmation purposes. This highlights the interest and potential of analytical techniques in the field of neuroprotection studies. The bioavailability and metabolic stability of polyphenols are areas that require further investigation. Additionally, the optimal dosing and delivery methods for polyphenols are yet to be established.

In conclusion, polyphenols represent a promising avenue for AD research, offering potential benefits in terms of neuroprotection and disease modulation. The integration of in vitro and in vivo studies, coupled with advances in analytical chemistry and chemometric tools, will be crucial in advancing our understanding of the role of polyphenols in AD. As we move forward, it will be essential to continue exploring these natural compounds, not only for their potential therapeutic benefits but also for their contribution to promoting overall well-being. The process of uncovering the full potential of polyphenols in AD treatment continues, and the current state of research holds the promise of thrilling advancements in the future.

Looking ahead, it is crucial to recognize the potential of computational tools such as molecular docking in neuroprotection research. These tools can offer valuable insights into the interactions between polyphenols and enzymes, Aβ_42_ and tau proteins and in vivo models, serving as a robust complement to experimental methods. Preliminary studies and initial compound screening can particularly benefit from these computational approaches, enabling the identification of promising compounds more efficiently. An increasing role is foreseen for these computational tools in enhancing the understanding of polyphenols and their neuroprotective effects. The integration of these tools in future research endeavors is strongly advocated, as they can provide a more comprehensive view of the potential of polyphenols in the treatment of AD.

## Figures and Tables

**Table 1:** Main groups of polyphenols and their representative sources from dietary and agri-food residues.

Phenolic Family	Dietary Sources	Agri-Food Residue or Non-Edible Sources
Phenolic acid		
Hydroxycinnamic acid	Almonds, cereals, cherries, citrus juices and fruits, coffee, corn flour, peaches, plums, potato, rice flour, spinach, tomatoes, and wheat flour.	Apple pomace, artichoke wastewaters, banana peel, citrus peels, olive mill wastewaters, and spent coffee grounds.
Hydroxybenzoic acid	Black currant, blackberry, cereals, coffee, cowpea, oilseeds, raspberry, and wheat flour.	Citrus peels, grape pomace, residual brewing yeast, squash shells and seeds.
Flavonoids		
Flavonols	Apples, apricot, arugula, beans, capers, cloves, leeks, lettuce, onions, saffron, and tomatoes.	Apples peels, banana peels, grape pomace and seeds, guava peels and seeds, onion peels, olive leaves and pomace.
Flavones	Artichoke, black olive, celery, citrus fruits, oregano, peanut, parsley, pepper, spinach.	Artichokes steams, camu-camu peels and peanut skin and shell
Isoflavones	Red clover, soybeans, soymilk and soy flour.	Soy processing waste and peanut skins and shells.
Flavanols	Grapes, apples, tomatoes, leeks, lettuces, curly kale, berries, onions, red grapes, beans, green and black, cider, tea, red wine.	Appel peel, grape seeds, peels and pomace and tea by-products.
Anthocyanins	Eggplant, grape, plums, pomegranate, raspberries, red and back-currants, red cabbage, red wine, strawberries, and radish.	Grape skins and seeds, grape pomace and floral tepals (saffron).
Flavanones	Citrus juices, citrus fruits, peppermint, fennel and rosemary.	Banana peels, citrus seeds, peels and pomace and residual brewing yeast.
Stilbenes	Almonds, grapes, red wine and berries.	Avocado peels, grape skins, seeds, pomace and stems.
Lignans	Broccoli, flaxseed, kale, lentil, sesame seeds, tea, wine and wheat.	Coffee, soybeans and wine residues.
Tannins		
Hydrolizable tannins	Pomegranates, raspberries and tea	Pomegranate peels and seeds and tea by-products.
Condense tannins	Apples, chestnut, grapes, pears, peaches, and hazelnuts.	Grape seeds
Phlorotannins	-	Brown seaweed
Complex tannins	-	Cork by-products (e.g., tree bark)

Sources: Alara et al. [14], Câmara et al. [29], Belščak-Cvitanović et al. [31], and Durazzo et al. [34].

**Table 2:** Extraction methodologies applied to the recovery of phenolic compounds from agricultural matrices for the assessment of their neurodegenerative potential.

Extraction Technique	Source (Amount)	Sample Treatment	Extraction Solvent (Volume)	Extraction Conditions	Major Polyphenols (Amount)	Ref.
Soxhlet	Grape leaves (*Vitis vinifera*) (40 g)	Air-dried and ground	Ethanol (not specified)	Extraction at 78–80 °C for 36 h, followed by filtration and solvent evaporation	Catechin and quercetin (55–197, mg·100 g^−1^)	[50]
	Pomegranate peels (*Punica granatum* L.) (not specified)	Air-dried and ground	Chloroform Ethyl acetate Butanol (not specified)	Extraction for 6 h at temperature above the boiling point, followed solvent evaporation	Total polyphenol (6–9 mg CAE·g^−1^)	[51]
Solid-liquid extraction	Lemon peel (*Citrus limon*) (1.0 g)	Freeze-dried and ground	Ethanol-Water 80:20 (*v*/*v*) (25 mL)	Stirring for 2 h min. Then the extract was centrifuged, filtered and the solvent was evaporated	Not specified	[52]
	Wild blueberry leaves (*Vaccinium carymbosum*) (1.0 g)	Dried with liquid nitrogen and ground	Acetone-Water 80:20 (*v*/*v*) acidified with 0.2% (*v*/*v*) formic acid (5 mL)	Vortex stirring and shaken on ice for 30 min, followed by centrifugation	Delphinidin and cyanidin glucosides (22–117 mg·100 g^−1^)	[53]
	Passion fruit seeds (*Passiflora edulis*) (5.0 g)	Oven-dried at 45 °C, ground and defatted	Ethanol-Water 79:21 (*v*/*v*) (30 mL)	Mechanical stirring for 80 min at 85 °C and 250 rpm, followed by centrifugation	Piceatannol (23.4 mg·g^−1^)	[54]
	Mulberry leaves (*Morus macrour*) (1.0 kg)	Dried	Ethanol-Water 80:20 (*v*/*v*) (500 mL)	Extraction was performed three consecutive times, then the solvent was evaporated	Resveratrol, chrysin, moracin D and ferulic acid (not specified)	[55]
	Olive mesocarp (*Olea europaea*) (3.0 g)	Freeze-dried and ground	Methanol (12 mL)	Vortex stirring for 1 min at 20 °C. Then the extract was centrifuged and lyophilized	Oleuropein and derivatives (not specified)	[56]
Maceration	Avocado leaves and seeds (*Persea americana* Mill.) (1.0 g)	Cut and ground	Water (100 mL)	Soaking the sample for 24 h, final filtration and centrifugation	Total polyphenol (92 mg GAE·g^−1^)	[57]
	Avocado peels and seeds (*Persea americana* Mill.) (50.0 and 100 g)	Dried, cut and ground	1st Hexane 2nd Ethanol (100 mL)	Fractionated extraction. The extractions were performed three times for 24 h. The solvent was removed in a rotary evaporator (60 °C)	Caffeic acid, (epi)catechin, rutin, B- type procyanidins (not specified)	[58]
	Orange peel (*Citrus sinensis*) (20 mg)	Oven-dried at 50 °C and ground	Water (200 mL)	Soaking the sample for 72 h at 37 °C, further filtration, solvent evaporation and freeze-dried of the extract	Gallic acid, quercetin, naringenin, propyl gallate and rutin (3.0–1.2, mg·100 g^−1^)	[59]
	Cranberry (*Vaccinium oxycoccos*) (500 g)	Air-dried and ground	Ethanol (2.5 L)	Soaking the sample for 48 h, followed by filtration and solvent evaporation	Cyanidin-, peonidin-, myricetin- and quercetin 3-*O*-glucoside (96–227, mg·100 g^−1^)	[60]
	Mastic leaves (*Pistacia lentiscus* L.) (150 g)	Air-dried and ground	Ethanol (600 mL)	Agitation for 24 h, followed by decantation, centrifugation, and solvent evaporation	Total polyphenol (95 g GAE·kg^−1^)	[61]
UAE	Cinnamon barks and buds (*Cinnamomum zeylanicum* and *Cinnamomum cassia*) (2.0 g)	Ground	Ethanol (20 mL)	Sonication in ultrasonic bath at 37 kHz, 45 °C for 60 min, followed by centrifugation, filtration, evaporation of the solvent and lyophilization	B-type procyanidins (not quantified)	[62]
	Spent coffee grounds (*Coffea arabica*) (10 g)	Oven-dried at 50 °C and ground	Methanol Water Methanol-Water 50:50 (*v*/*v*) Ethanol-Water 70:30 (*v*/*v*) (50 mL)	Sonication in ultrasonic bath at 40 kHz, 20 °C for 120 min, followed by filtration and lyophilization	5-caffeoylquinic acid and 3,5-dicaffeoylquinic acid (52–0.9 mg·g^−1^)	[63]
	Moss (*Lycopodium selago*) (1.0 g)	Dried and crushed	Ethanol-Water 70:30 (*v*/*v*) (30 mL)	Ultrasonic probe sonication at 20 kHz and 70% amplitude, followed by centrifugation and lyophilization	Total polyphenol (9.21 mg GAE·g^−1^)	[64]
	Mushrooms (0.2 g)	Freeze-dried and crushed	Methanol-Water 93.6:6.4 (*v*/*v*) (15.3 mL)	Ultrasonic probe sonication at 20 Hz, 70 W, 16.86% amplitude, 0.71 s^−1^ cycles and 60 °C for 5 min, followed by centrifugation	Total polyphenol (10–13, mg GAE·g^−1^)	[65]
PLE	Passion fruit seeds (*Passiflora edulis*) (1.0 g)	Oven-dried at 45 °C, ground and defatted	Ethanol (25 mL)	Sample mixing with 1.0 g of sea sand and extraction at 110 °C and 1500 psi for 20 min, with 3 cycles	Piceatannol (56.5 mg·g^−1^)	[54]
	Peach (*Prunus persica*) (1.0 g)	Freeze-dried at 45 °C and ground	Ethanol-Water 50:50 (*v*/*v*) (no specified)	Sample mixing with 1.0 g of sea sand and extraction at 180 °C and 10 MPa for 5 min. The solvent was evaporated, and the extract was then freeze-dried	Total polyphenol (100 mg GAE·g^−1^)	[66]
	Tamarillo peels (*Cyphomandra betacea* (Cav.) Sendt) (1.0 g)	Freeze-dried at 45 °C and ground	Ethanol (no specified)	Extraction at 180 °C and 1500 psi for 20 min, followed by centrifugation	Hydroxycinnamic acid derivatives (not quantified)	[67]
EAE	Sweet cherry pomace (*Prunus avium*) (0.38 g)	Ground	Phosphate buffer 100 mM (1 mL)	Extraction was performed at 70 °C, pH = 10 for 40 min with 137.4 µL·g^−1^ Promod enzyme	(Epi)catechin (not quantified)	[68]
MSPD	Grape seeds (0.1 g)	Defatted by cold pressing	EtOH-Water 80:20 (*v*/*v*) (15 mL)	The sample was blended for 2 min with 0.5 g of diatomaceous earth and stirred (US bath) with 2 mL of the solvent for 5 min. Ultimately the extract was centrifuged	Catechin, gallic acid and dihydroxibenzoic acid (140–295, mg·kg^−1^)	[69]

**Table 3:** Main phenolic compounds used in AD in vitro studies and their observed mechanisms.

Phenolic Compounds	Family	Source	Mechanisms	References
Quercetin and apigenin	Flavonoid	Pure standards	Reduced Aβ_40_ aggregation, elongation rate and fibrils lenght. Generation of amorphous aggregates. AChE inhibition.	[155]
Catechin and epicatechin- derived adducts	Flavonoid	Pure standards	Reduced tau aggregation Increased neurites number and length in Neuro-2a cells.	[156]
Cannflavin A	Flavonoid	Pure standard	Reduction in Aβ_42_ fibrillation, density of aggregates. Binding with Aβ_42_ oligomes. Reduced Aβ_42_ induced cytotoxicity in PC12 cells	[130]
Mimulone	Flavonoid	Pure standard	Reduction in Aβ_42_ aggregates density. Reduced Aβ_42_ induced cytotoxicity in PC12 cells	[130]
Diplacone	Flavonoid	Pure standard	Reduction in Aβ_42_ fibrillation, density of aggregates. Reduced Aβ_42_ induced cytotoxicity in PC12 cells.	[130]
Amentoflavone	Biflavonoid	Pure standard	Inhibition of Aβ_42_ aggregation and disaggregation of preformed fibrils. Generation of amorphous Aβ_42_ aggregates	[157]
Caffeic acid Quercetin	Hydroxycinamic acid Flavonoid	*Jasonia glutinosa* (L.)	AChE, MAO-A and TYR inhibition	[112]
Gallic acid catechin	Hydroxybenzoic acid Flavonoid	*Desmodium elegans* DC.	AChE and BChE inhibition	[122]
Epigallocatechin gallate	Flavonoid	Pure standard	Protected against Aβ induced apoptosis, ROS and cytotoxicity in SH-SY5Y cells.	[158]
Resveratrol	Stilbene	Pure standard	Reduced tau expression and hyperphosphorilation in C6 cells. Reduced APP amyloidogenic pathway.	[159]
Punicalagin	Tannin	Pure standard	Reduced LPS induced cytotoxicity and neuroinflammation in BV2 cells. Reduced LPS-induced neuroinflammation and increased synaptic density in Neuro-2a cells	[160]
Tannic acid-loaded liposomes	Tannin	Pure standard	Reduced intracellular tau aggregation in SK-N-SH cells	[161]
Synapic acid	Hydroxycinamic acid	*Arabidopsis thaliana*	Reduced Aβ_25–35_ induced cytotoxicity and pro-inflamatory effect on BV2 cells	[162]

**Table 4:** Polyphenols used in the therapy in animal models of Alzheimer’s disease.

Animal Model	Description	Polyphenol or Extract	Studies in Animals	Behavioral Improvements	Effects on Brain	Adverse Effects	Ref.
APP/PS1	Transgenic	SeNPs coated with dihydromyricetin	Spatial learning ability: Morris Water Maze	Number of times they pass through the platform area	Overexpression and release of cytokines		[164]
Female Tg2576 mice	Transgenic	Rosmarinic acid	DNA microarray analysis in the brain, monoamine concentration in the brain, mRNA measurement in cerebral cortex		Increases monoamine neurotransmitters level		[163]
Male C57BL/6J mice	AD model induced D-gal 150 mg kg^−1^ day for 8 weeks	PU	Behavioral (Morris water maze, Y maze, open field test), brain Immunohistochemistry	Number of times they pass through the platform/rodents prefer to investigate a new arm	Increased survival neurons/improvement inflammatory process		[160]
Male Sprague Dawley rats	AD model induced Aluminum chloride 70 mg kg^−1^, for six weeks	*Opuntia ficus*-indica cladode, peel and fruit pulp extracts	Learning and memory functions (passive avoidance). Total antioxidant capacity in blood. Monoamine neurotransmitters in brain	number of times they avoid the chamber in which they will suffer shocks	Increases monoamines neurotransmitters level	>2000 mg kg^−1^ body weight	[126]
Male albinos mice	AD model induced Aluminum chloride 100 ppm day for two months	*P. lentiscus* L. leaves extract	Behavioral (anxiety: head-dipping, black and white and elevated plus maze and memory: Morris water maze) histological (brain pathological changes)	Number of times they pass through the platform area	Protective effects in oxidative stress and lipid peroxidation		[61]
Mice	AD model induced scopolamine at a dose of 1 mg kg^−1^ for up to 9 days	Crude extracts of *Desmodium elegans*	Behavioral (Water maze, Y maze, elevated plus maze)	Number of times they pass through the platform area/rodents prefer to investigate a new arm			[122]
C57BL/6 naive mice	Single intracerebroventricular (ICV) injection of Aβ Oligomers	Natural-based complex polyphenols	NOR. Memory task	Improvements in recognition of new objects	Reduction on microglial activation		[165]
Male SAMP8 mice	Model of age-related cognitive decline with relevance to alterations of the gene expression and protein abnormalities in AD	Sugarcane (*Saccharum officinarum* L.) Top Extract	Behavioral (Morris water maze), brain Immunohistochemistry, monoamine concentration in the brain, cRNA measurement in cerebral cortex	Number of times they pass through the platform area	Increases monoamines neurotransmitters level		[129]
Female NMRI mice	Aging model	Olive secoiridoid polyphenols	Behavioral (Passive Avoidance, Y maze). ATP levels	latency to cross through the gate between compartments/rodents prefer to investigate a new arm	Increases ATP levels		[123]
*Caernorhabditis elegans*	CL4176 transgenic sensible to temperature (25 °C), 5-hydroxytryptamine hypersensitivity	Ethyl caffeate	Paralysis symptoms	delayed the paralysis symptoms	Increases monoamines neurotransmitters level	>600 µM	[166]
*Caenorhabditis elegans*	Transgenic strain (CL4176 (smg-1 ts 131 (myo-3/Aβ_42_ long 30-UTR)) temperature-sensitive transgene	*Jasonia glutinosa* extract	Paralysis symptoms	delayed the paralysis symptoms	Increases monoamines neurotransmitters level		[112]
*Caenorhabditis elegans*	CL2006 transgenic. expressing the Aβ_3-42_ peptide	Hop Extracts (*Humulus lupulus* L.)	Mobility Assay	Paralysis reduction			[128]
*Drosophila* flies	*Drosophila melanogaster* model	Extract of *Arabidopsis thaliana*	Locomotor dysfunction induced by Aβ_42_	Improvement climbed ability	Anti-inflammatory		[162]
*Drosophila* flies	*Drosophila melanogaster* model	p-Coumaric acid	*Drosophila* lines (GMR-Aβ_42_ for the eye assay and Actin5C-Aβ42 for the lifespan and locomotive assays)				[114]
Zebrafish	Scopolamine-Induced Memory Deficits	Extract of *Lycopodium selago* L.	Spatial Memory in Y-Maze, novel tank-diving, NOR				[64]

## Abbreviations

ACh, acetylcholine; AChE, acetylcholinesterase; AD, Alzheimer’s disease; AFM, atomic force microscopy; ANOVA, analysis of variance; APP, amyloid precursor protein; Aβ, amyloid-beta; BBB, blood-brain barrier; BChE, butyrylcholinesterase; CAT-PhG, catechin-phloroglucinol; CAT-PYR, catechin-pyrogallol; CE, capillary electrophoresis; cLC, capillary liquid chromatography; CNS, central nervous system; DAD, diode array detection; DCF, dichlorofluorescein; DLS, dynamic light scattering; DMAC, p-dimethylaminocinnamaldehyde; EAE, enzyme-assisted extraction; EGCG, epigallocatechin gallate; EPIC-PYR, epicatechin-pyrogallol; ESI, electrospray ionization; FTIR, Fourier-transform infrared spectroscopy; GA, gallic acid; GC, gas chromatography; HILIC, hydrophilic interaction liquid chromatography; HPLC, high-performance liquid chromatography; IC_50_, half maximal inhibitory concentration; LC, liquid chromatography; LPS, lipopolysaccharide; LSD, least significant difference; MAO-A, monoamine oxidase A; MDA, malondialdehyde; MS, mass spectrometry; MS/MS, tandem mass spectrometry; MSPD, matrix solid-phase dispersion extraction; MTT, 3-(4,5-dimethylthiazol-2-yl)-2,5-diphenyltetrazolium bromide; NFTs, neurofibrillary tangles; NMR, nuclear magnetic resonance; NOR, novel object recognition; PCA, principal component analysis; PLE, pressurized liquid extraction; PT_50_; half maximal paralyzed concentration; PU, punicalagin; RNS, reactive nitrogen species; ROS, reactive oxygen species; RP, reversed phase; SFC, supercritical fluid chromatography; SLE, solid-liquid extraction; SPE, solid-phase extraction; TA, tannic acid; TEM, transmission electron microscopy; TFC, total flavonoid content; ThS, thioflavin S; ThT, thioflavin T; TLC, thin-layer chromatography; TMA, total monomeric anthocyanins; TOF, time-of-flight; TPC, total phenolic content; UAE, ultrasound-assisted extraction; UHPLC, ultrahigh performance liquid chromatography.

## References

[1] DeTure M.A., Dickson D.W. (2019). The Neuropathological Diagnosis of Alzheimer’s Disease. Mol. Neurodegener..

[2] Shin J.-H. (2022). Dementia Epidemiology Fact Sheet 2022. Ann. Rehabil. Med..

[3] Joana Gil-Chávez G., Villa J.A., Fernando Ayala-Zavala J., Basilio Heredia J., Sepulveda D., Yahia E.M., González-Aguilar G.A. (2013). Technologies for Extraction and Production of Bioactive Compounds to Be Used as Nutraceuticals and Food Ingredients: An Overview. Compr. Rev. Food Sci. Food Saf..

[4] Bié J., Sepodes B., Fernandes P.C.B., Ribeiro M.H.L. (2023). Polyphenols in Health and Disease: Gut Microbiota, Bioaccessibility, and Bioavailability. Compounds.

[5] Plamada D., Vodnar D.C. (2021). Polyphenols—Gut Microbiota Interrelationship: A Transition to a New Generation of Prebiotics. Nutrients.

[6] Silva-Weiss A., Ihl M., Sobral P.J.A., Gómez-Guillén M.C., Bifani V. (2013). Natural Additives in Bioactive Edible Films and Coatings: Functionality and Applications in Foods. Food Eng. Rev..

[7] Guuaadaoui A., Benaicha S., Elmajdoui N., Bellaoui M., Hamal A. (2014). What Is a Bioactive Compound? A Combined Definition for a Preliminary Consensus. Int. J. Nutr. Food Sci..

[8] Fernandes S.S., Coelho M.S., Salas-Mellado M.D.L.M. (2018). Bioactive Compounds as Ingredients of Functional Foods: Polyphenols, Carotenoids, Peptides from Animal and Plant Sources New.

[9] Scarano A., Laddomada B., Blando F., De Santis S., Verna G., Chieppa M., Santino A. (2023). The Chelating Ability of Plant Polyphenols Can Affect Iron Homeostasis and Gut Microbiota. Antioxidants.

[10] Yahfoufi N., Alsadi N., Jambi M., Matar C. (2018). The Immunomodulatory and Anti-Inflammatory Role of Polyphenols. Nutrients.

[11] Pritam P., Deka R., Bhardwaj A., Srivastava R., Kumar D., Jha A.K., Jha N.K., Villa C., Jha S.K. (2022). Antioxidants in Alzheimer’s Disease: Current Therapeutic Significance and Future Prospects. Biology.

[12] Arias-Sánchez R.A., Torner L., Fenton Navarro B. (2023). Polyphenols and Neurodegenerative Diseases: Potential Effects and Mechanisms of Neuroprotection. Molecules.

[13] Dias A.L.B., de Aguiar A.C., Rostagno M.A. (2021). Extraction of Natural Products Using Supercritical Fluids and Pressurized Liquids Assisted by Ultrasound: Current Status and Trends. Ultrason. Sonochem..

[14] Alara O.R., Abdurahman N.H., Ukaegbu C.I. (2021). Extraction of Phenolic Compounds: A Review. Curr. Res. Food Sci..

[15] Cassani L., Gomez-Zavaglia A. (2022). Sustainable Food Systems in Fruits and Vegetables Food Supply Chains. Front. Nutr..

[16] Shi L., Zhao W., Yang Z., Subbiah V., Suleria H.A.R. (2022). Extraction and Characterization of Phenolic Compounds and Their Potential Antioxidant Activities. Environ. Sci. Pollut. Res..

[17] Sun M.-F., Jiang C.-L., Kong Y.-S., Luo J.-L., Yin P., Guo G.-Y. (2022). Recent Advances in Analytical Methods for Determination of Polyphenols in Tea: A Comprehensive Review. Foods.

[18] Yang W., Cui K., Li X., Zhao J., Zeng Z., Song R., Qi X., Xu W. (2021). Effect of Polyphenols on Cognitive Function: Evidence from Population-Based Studies and Clinical Trials. J. Nutr. Health Aging.

[19] Prasanna P., Upadhyay A. (2021). Flavonoid-Based Nanomedicines in Alzheimer’s Disease Therapeutics: Promises Made, a Long Way To Go. ACS Pharmacol. Transl. Sci..

[20] Vicente-Zurdo D., Rosales-Conrado N., León-González M.E. (2024). Unravelling the In Vitro and In Vivo Potential of Selenium Nanoparticles in Alzheimer’s Disease: A Bioanalytical Review. Talanta.

[21] Fakhri S., Abbaszadeh F., Moradi S.Z., Cao H., Khan H., Xiao J. (2022). Effects of Polyphenols on Oxidative Stress, Inflammation, and Interconnected Pathways during Spinal Cord Injury. Oxid. Med. Cell. Longev..

[22] Li Z., Zhao T., Shi M., Wei Y., Huang X., Shen J., Zhang X., Xie Z., Huang P., Yuan K. (2023). Polyphenols: Natural Food Grade Biomolecules for Treating Neurodegenerative Diseases from a Multi-Target Perspective. Front. Nutr..

[23] Gentile M.T., Camerino I., Ciarmiello L., Woodrow P., Muscariello L., De Chiara I., Pacifico S. (2023). Neuro-Nutraceutical Polyphenols: How Far Are We?. Antioxidants.

[24] Grabska-Kobyłecka I., Szpakowski P., Król A., Książek-Winiarek D., Kobyłecki A., Głąbiński A., Nowak D. (2023). Polyphenols and Their Impact on the Prevention of Neurodegenerative Diseases and Development. Nutrients.

[25] Kalogiouri N.P., Aalizadeh R., Dasenaki M.E., Thomaidis N.S. (2020). Application of High Resolution Mass Spectrometric Methods Coupled with Chemometric Techniques in Olive Oil Authenticity Studies—A Review. Anal. Chim. Acta.

[26] Granato D., Santos J.S., Escher G.B., Ferreira B.L., Maggio R.M. (2018). Use of Principal Component Analysis (PCA) and Hierarchical Cluster Analysis (HCA) for Multivariate Association between Bioactive Compounds and Functional Properties in Foods: A Critical Perspective. Trends Food Sci. Technol..

[27] Chiriac E., Chiţescu C., Geană E.-I., Gird C., Socoteanu R., Boscencu R. (2021). Advanced Analytical Approaches for the Analysis of Polyphenols in Plants Matrices—A Review. Separations.

[28] Feizi N., Hashemi-Nasab F.S., Golpelichi F., Saburouh N., Parastar H. (2021). Recent Trends in Application of Chemometric Methods for GC-MS and GC×GC-MS-Based Metabolomic Studies. TrAC Trends Anal. Chem..

[29] Câmara J.S., Albuquerque B.R., Aguiar J., Corrêa R.C.G., Gonçalves J.L., Granato D., Pereira J.A.M., Barros L., Ferreira I.C.F.R. (2021). Food Bioactive Compounds and Emerging Techniques for Their Extraction: Polyphenols as a Case Study. Foods.

[30] Abbas M., Saeed F., Anjum F.M., Afzaal M., Tufail T., Bashir M.S., Ishtiaq A., Hussain S., Suleria H.A.R. (2017). Natural Polyphenols: An Overview. Int. J. Food Prop..

[31] Belščak-Cvitanović A., Durgo K., Huđek A., Bačun-Družina V., Komes D. (2018). Overview of Polyphenols and Their Properties. Polyphenols: Properties, Recovery, and Applications.

[32] di Ferdinando M., Brunetti C., Agati G., Tattini M. (2014). Multiple Functions of Polyphenols in Plants Inhabiting Unfavorable Mediterranean Areas. Environ. Exp. Bot..

[33] Tsao R. (2010). Chemistry and Biochemistry of Dietary Polyphenols. Nutrients.

[34] Durazzo A., Lucarini M., Souto E.B., Cicala C., Caiazzo E., Izzo A.A., Novellino E., Santini A. (2019). Polyphenols: A Concise Overview on the Chemistry, Occurrence, and Human Health. Phytother. Res..

[35] Manach C., Scalbert A., Morand C., Rémésy C., Jiménez L. (2004). Polyphenols: Food Sources and Bioavailability. Am. J. Clin. Nutr..

[36] Ignat I., Volf I., Popa V.I. (2011). A Critical Review of Methods for Characterisation of Polyphenolic Compounds in Fruits and Vegetables. Food Chem..

[37] Esparza I., Jiménez-Moreno N., Bimbela F., Ancín-Azpilicueta C., Gandía L.M. (2020). Fruit and Vegetable Waste Management: Conventional and Emerging Approaches. J. Environ. Manag..

[38] de Albuquerque B.R., Corrêa R.C.G., de Lima Sampaio S., Barros L. (2023). Bioactive Compounds from Food and Its By-Products: Current Applications and Future Perspectives.

[39] Drevelegka I., Goula A.M. (2020). Recovery of Grape Pomace Phenolic Compounds through Optimized Extraction and Adsorption Processes. Chem. Eng. Process.-Process Intensif..

[40] Banerjee J., Singh R., Vijayaraghavan R., MacFarlane D., Patti A.F., Arora A. (2017). Bioactives from Fruit Processing Wastes: Green Approaches to Valuable Chemicals. Food Chem..

[41] Shahidi F., Ambigaipalan P. (2015). Phenolics and Polyphenolics in Foods, Beverages and Spices: Antioxidant Activity and Health Effects—A Review. J. Funct. Foods.

[42] Haminiuk C.W.I., Maciel G.M., Plata-Oviedo M.S.V., Peralta R.M. (2012). Phenolic Compounds in Fruits—An Overview. Int. J. Food Sci. Technol..

[43] Xie Y., Chen J., Xiao A., Liu L. (2017). Antibacterial Activity of Polyphenols: Structure-Activity Relationship and Influence of Hyperglycemic Condition. Molecules.

[44] de Araújo F.F., de Paulo Farias D., Neri-Numa I.A., Pastore G.M. (2021). Polyphenols and Their Applications: An Approach in Food Chemistry and Innovation Potential. Food Chem..

[45] Farhadi F., Khameneh B., Iranshahi M., Iranshahy M. (2019). Antibacterial Activity of Flavonoids and Their Structure–Activity Relationship: An Update Review. Phytother. Res..

[46] Roberfroid M.B. (2002). Functional Foods: Concepts and Application to Inulin and Oligofructose. Br. J. Nutr..

[47] Albuquerque B.R., Heleno S.A., Oliveira M.B.P.P., Barros L., Ferreira I.C.F.R. (2021). Phenolic Compounds: Current Industrial Applications, Limitations and Future Challenges. Food Funct..

[48] de Lima Cherubim D.J., Buzanello Martins C.V., Oliveira Fariña L., da Silva de Lucca R.A. (2020). Polyphenols as Natural Antioxidants in Cosmetics Applications. J. Cosmet. Dermatol..

[49] Sagar N.A., Pareek S., Sharma S., Yahia E.M., Lobo M.G. (2018). Fruit and Vegetable Waste: Bioactive Compounds, Their Extraction, and Possible Utilization. Compr. Rev. Food Sci. Food Saf..

[50] Duangjan C., Rangsinth P., Zhang S., Gu X., Wink M., Tencomnao T. (2021). Vitis Vinifera Leaf Extract Protects Against Glutamate-Induced Oxidative Toxicity in HT22 Hippocampal Neuronal Cells and Increases Stress Resistance Properties in Caenorhabditis Elegans. Front. Nutr..

[51] Khokar R., Hachani K., Hasan M., Othmani F., Essam M., Al Mamari A., UM D., Khan S.A. (2021). Anti-Alzheimer Potential of a Waste by-Product (Peel) of Omani Pomegranate Fruits: Quantification of Phenolic Compounds, in-Vitro Antioxidant, Anti-Cholinesterase and in-Silico Studies. Biocatal. Agric. Biotechnol..

[52] Arcone R., D’Errico A., Nasso R., Rullo R., Poli A., Di Donato P., Masullo M. (2023). Inhibition of Enzymes Involved in Neurodegenerative Disorders and Aβ1–40 Aggregation by Citrus Limon Peel Polyphenol Extract. Molecules.

[53] Debnath-Canning M., Unruh S., Vyas P., Daneshtalab N., Igamberdiev A.U., Weber J.T. (2020). Fruits and Leaves from Wild Blueberry Plants Contain Diverse Polyphenols and Decrease Neuroinflammatory Responses in Microglia. J. Funct. Foods.

[54] Dos Santos L.C., Mendiola J.A., Sánchez-camargo A.D.P., Álvarez-rivera G., Viganó J., Cifuentes A., Ibáñez E., Martínez J. (2021). Selective Extraction of Piceatannol from Passiflora Edulis By-products: Application of Hsps Strategy and Inhibition of Neurodegenerative Enzymes. Int. J. Mol. Sci..

[55] El-Hawary S.S., Sayed A.M., Issa M.Y., Ebrahim H.S., Alaaeldin R., Elrehany M.A., Abd El-Kadder E.M., Abdelmohsen U.R. (2021). Anti-Alzheimer Chemical Constituents of Morus Macroura Miq.: Chemical Profiling, In Silico and In Vitro Investigations. Food Funct..

[56] Mohammad-Beigi H., Aliakbari F., Sahin C., Lomax C., Tawfike A., Schafer N.P., Amiri-Nowdijeh A., Eskandari H., Møller I.M., Hosseini-Mazinani M. (2019). Oleuropein Derivatives from Olive Fruit Extracts Reduce α-Synuclein Fibrillation and Oligomer Toxicity. J. Biol. Chem..

[57] Oboh G., Odubanjo V.O., Bello F., Ademosun A.O., Oyeleye S.I., Nwanna E.E., Ademiluyi A.O. (2016). Aqueous Extracts of Avocado Pear (*Persea americana* Mill.) Leaves and Seeds Exhibit Anti-Cholinesterases and Antioxidant Activities In Vitro. J. Basic. Clin. Physiol. Pharmacol..

[58] da Silva G.G., Pimenta L.P.S., Melo J.O.F., Mendonça H.d.O.P., Augusti R., Takahashi J.A. (2022). Phytochemicals of Avocado Residues as Potential Acetylcholinesterase Inhibitors, Antioxidants, and Neuroprotective Agents. Molecules.

[59] Abd El-Aziz N.M., Shehata M.G., Alsulami T., Badr A.N., Elbakatoshy M.R., Ali H.S., El-Sohaimy S.A. (2023). Characterization of Orange Peel Extract and Its Potential Protective Effect against Aluminum Chloride-Induced Alzheimer’s Disease. Pharmaceuticals.

[60] Balawejder M., Piechowiak T., Kapusta I., Chęciek A., Matłok N. (2023). In Vitro Analysis of Selected Antioxidant and Biological Properties of the Extract from Large-Fruited Cranberry Fruits. Molecules.

[61] Azib L., Debbache-Benaida N., Da Costa G., Atmani-Kilani D., Saidene N., Ayouni K., Richard T., Atmani D. (2019). *Pistacia lentiscus* L. Leaves Extract and Its Major Phenolic Compounds Reverse Aluminium-Induced Neurotoxicity in Mice. Ind. Crops Prod..

[62] Ciaramelli C., Palmioli A., Angotti I., Colombo L., De Luigi A., Sala G., Salmona M., Airoldi C. (2022). NMR-Driven Identification of Cinnamon Bud and Bark Components with Anti-Aβ Activity. Front. Chem..

[63] Angeloni S., Freschi M., Marrazzo P., Hrelia S., Beghelli D., Juan-García A., Juan C., Caprioli G., Sagratini G., Angeloni C. (2021). Antioxidant and Anti-Inflammatory Profiles of Spent Coffee Ground Extracts for the Treatment of Neurodegeneration. Oxid. Med. Cell. Longev..

[64] Valu M.V., Ducu C., Moga S., Negrea D., Hritcu L., Boiangiu R.S., Vamanu E., Balseanu T.A., Carradori S., Soare L.C. (2021). Effects of the Hydroethanolic Extract of *Lycopodium selago* L. On Scopolamine-Induced Memory Deficits in Zebrafish. Pharmaceuticals.

[65] Aliaño-González M.J., Barea-Sepúlveda M., Espada-Bellido E., Ferreiro-González M., López-Castillo J.G., Palma M., Barbero G.F., Carrera C. (2022). Ultrasound-Assisted Extraction of Total Phenolic Compounds and Antioxidant Activity in Mushrooms. Agronomy.

[66] Guo C., Valdés A., Sánchez-Martínez J.D., Ibáñez E., Bi J., Cifuentes A. (2022). Neuroprotective Potential of Thinned Peaches Extracts Obtained by Pressurized Liquid Extraction after Different Drying Processes. Foods.

[67] Suárez-Montenegro Z.J., Ballesteros-Vivas D., Gallego R., Valdés A., Sánchez-Martínez J.D., Parada-Alfonso F., Ibáñez E., Cifuentes A. (2021). Neuroprotective Potential of Tamarillo (*Cyphomandra betacea*) Epicarp Extracts Obtained by Sustainable Extraction Process. Front. Nutr..

[68] Domínguez-Rodríguez G., Ramón Vidal D., Martorell P., Plaza M., Marina M.L. (2022). Composition of Nonextractable Polyphenols from Sweet Cherry Pomace Determined by DART-Orbitrap-HRMS and Their In Vitro and In Vivo Potential Antioxidant, Antiaging, and Neuroprotective Activities. J. Agric. Food Chem..

[69] Gómez-Mejía E., Vicente-Zurdo D., Rosales-Conrado N., León-González M.E., Madrid Y. (2022). Screening the Extraction Process of Phenolic Compounds from Pressed Grape Seed Residue: Towards an Integrated and Sustainable Management of Viticultural Waste. LWT.

[70] Zhou Y., Xu X.Y., Gan R.Y., Zheng J., Li Y., Zhang J.J., Xu D.P., Li H. (2019). bin Optimization of Ultrasound-Assisted Extraction of Antioxidant Polyphenols from the Seed Coats of Red Sword Bean (*Canavalia gladiate* (Jacq.) DC.). Antioxidants.

[71] Tapia-Quirós P., Montenegro-Landívar M.F., Reig M., Vecino X., Alvarino T., Cortina J.L., Saurina J., Granados M. (2020). Olive Mill and Winery Wastes as Viable Sources of Bioactive Compounds: A Study on Polyphenols Recovery. Antioxidants.

[72] Montenegro-Landívar M.F., Tapia-Quirós P., Vecino X., Reig M., Valderrama C., Granados M., Cortina J.L., Saurina J. (2021). Recovery of Added-Value Compounds from Orange and Spinach Processing Residues: Green Extraction of Phenolic Compounds and Evaluation of Antioxidant Activity. Antioxidants.

[73] Roselló-Soto E., Koubaa M., Moubarik A., Lopes R.P., Saraiva J.A., Boussetta N., Grimi N., Barba F.J. (2015). Emerging Opportunities for the Effective Valorization of Wastes and By-Products Generated during Olive Oil Production Process: Non-Conventional Methods for the Recovery of High-Added Value Compounds. Trends Food Sci. Technol..

[74] Ameer K., Shahbaz H.M., Kwon J.H. (2017). Green Extraction Methods for Polyphenols from Plant Matrices and Their Byproducts: A Review. Compr. Rev. Food Sci. Food Saf..

[75] Žlabur J., Voća S., Brnčić M., Rimac-Brnčić S. (2018). New Trends in Food Technology for Green Recovery of Bioactive Compounds From Plant Materials. Role of Materials Science in Food Bioengineering.

[76] Wianowska D., Gil M. (2019). New Insights into the Application of MSPD in Various Fields of Analytical Chemistry. TrAC Trends Anal. Chem..

[77] Gómez-Mejía E., Mikkelsen L.H., Rosales-Conrado N., León-González M.E., Madrid Y. (2021). A Combined Approach Based on Matrix Solid-Phase Dispersion Extraction Assisted by Titanium Dioxide Nanoparticles and Liquid Chromatography to Determine Polyphenols from Grape Residues. J. Chromatogr. A.

[78] Capriotti A.L., Cavaliere C., Laganà A., Piovesana S., Samperi R. (2013). Recent Trends in Matrix Solid-Phase Dispersion. TrAC Trends Anal. Chem..

[79] Tomaz I., Huzanić N., Preiner D., Stupić D., Andabaka Ž., Maletić E., Kontić J.K., Ašperger D. (2019). Extraction Methods of Polyphenol from Grapes: Extractions of Grape Polyphenols. Polyphenols in Plants.

[80] Di Donato P., Taurisano V., Tommonaro G., Pasquale V., Jiménez J.M.S., de Pascual-Teresa S., Poli A., Nicolaus B. (2018). Biological Properties of Polyphenols Extracts from Agro Industry’s Wastes. Waste Biomass Valorization.

[81] Gómez-Mejía E., Rosales-Conrado N., León-González M.E., Madrid Y. (2019). Determination of Phenolic Compounds in Residual Brewing Yeast Using Matrix Solid-Phase Dispersion Extraction Assisted by Titanium Dioxide Nanoparticles. J. Chromatogr. A.

[82] Castillo A., Celeiro M., Rubio L., Bañobre A., Otero-Otero M., Garcia-Jares C., Lores M. (2022). Optimization of Bioactives Extraction from Grape Marc via a Medium Scale Ambient Temperature System and Stability Study. Front. Nutr..

[83] Lucci P., Saurina J., Núñez O. (2017). Trends in LC-MS and LC-HRMS Analysis and Characterization of Polyphenols in Food. TrAC Trends Anal. Chem..

[84] Khoddami A., Wilkes M., Roberts T. (2013). Techniques for Analysis of Plant Phenolic Compounds. Molecules.

[85] Aleixandre-Tudo J.L., Buica A., Nieuwoudt H., Aleixandre J.L., du Toit W. (2017). Spectrophotometric Analysis of Phenolic Compounds in Grapes and Wines. J. Agric. Food Chem..

[86] Razem M., Ding Y., Morozova K., Mazzetto F., Scampicchio M. (2022). Analysis of Phenolic Compounds in Food by Coulometric Array Detector: A Review. Sensors.

[87] Sánchez-Rangel J.C., Benavides J., Heredia J.B., Cisneros-Zevallos L., Jacobo-Velázquez D.A. (2013). The Folin–Ciocalteu Assay Revisited: Improvement of Its Specificity for Total Phenolic Content Determination. Anal. Methods.

[88] Carrasco-Pancorbo A., Cerretani L., Bendini A., Segura-Carretero A., Gallina-Toschi T., Fernández-Gutiérrez A. (2005). Analytical Determination of Polyphenols in Olive Oils. J. Sep. Sci..

[89] Ma S., Kim C., Neilson A.P., Griffin L.E., Peck G.M., O’Keefe S.F., Stewart A.C. (2019). Comparison of Common Analytical Methods for the Quantification of Total Polyphenols and Flavanols in Fruit Juices and Ciders. J. Food Sci..

[90] Sik B., Székelyhidi R., Lakatos E., Kapcsándi V., Ajtony Z. (2022). Analytical Procedures for Determination of Phenolics Active Herbal Ingredients in Fortified Functional Foods: An Overview. Eur. Food Res. Technol..

[91] Margraf T., Karnopp A.R., Rosso N.D., Granato D. (2015). Comparison between Folin-Ciocalteu and Prussian Blue Assays to Estimate The Total Phenolic Content of Juices and Teas Using 96-Well Microplates. J. Food Sci..

[92] Mammen D., Daniel M. (2012). A Critical Evaluation on the Reliability of Two Aluminum Chloride Chelation Methods for Quantification of Flavonoids. Food Chem..

[93] Vijayalaxmi S., Jayalakshmi S.K., Sreeramulu K. (2015). Polyphenols from Different Agricultural Residues: Extraction, Identification and Their Antioxidant Properties. J. Food Sci. Technol..

[94] Naczk M., Shahidi F. (2004). Extraction and Analysis of Phenolics in Food. J. Chromatogr. A.

[95] Vidal-Casanella O., Núñez O., Granados M., Saurina J., Sentellas S. (2021). Analytical Methods for Exploring Nutraceuticals Based on Phenolic Acids and Polyphenols. Appl. Sci..

[96] Osman M., Mohd Hassan N., Khatib A., Tolos S. (2018). Antioxidant Activities of *Dialium indum* L. Fruit and Gas Chromatography-Mass Spectrometry (GC-MS) of the Active Fractions. Antioxidants.

[97] Ifeanacho M.O., Ikewuchi C.C., Ikewuchi J.C. (2017). Investigation of the Profile of Phenolic Compounds in the Leaves and Stems of Pandiaka Heudelotii Using Gas Chromatography Coupled with Flame Ionization Detector. Food Sci. Nutr..

[98] Motilva M.-J., Serra A., Macià A. (2013). Analysis of Food Polyphenols by Ultra High-Performance Liquid Chromatography Coupled to Mass Spectrometry: An Overview. J. Chromatogr. A.

[99] Žuvela P., Skoczylas M., Jay Liu J., Ba̧czek T., Kaliszan R., Wong M.W., Buszewski B. (2019). Column Characterization and Selection Systems in Reversed-Phase High-Performance Liquid Chromatography. Chem. Rev..

[100] Pyrzynska K., Sentkowska A. (2019). Chromatographic Analysis of Polyphenols. Polyphenols in Plants.

[101] Azaroual L., Liazid A., Mansouri F.E., Brigui J., Ruíz-Rodriguez A., Barbero G.F., Palma M. (2021). Optimization of the Microwave-Assisted Extraction of Simple Phenolic Compounds from Grape Skins and Seeds. Agronomy.

[102] Gómez-Mejía E., Rosales-Conrado N., León-González M.E., Madrid Y. (2019). Citrus Peels Waste as a Source of Value-Added Compounds: Extraction and Quantification of Bioactive Polyphenols. Food Chem..

[103] López-Fernández O., Domínguez R., Pateiro M., Munekata P.E.S., Rocchetti G., Lorenzo J.M. (2020). Determination of Polyphenols Using Liquid Chromatography–Tandem Mass Spectrometry Technique (LC–MS/MS): A Review. Antioxidants.

[104] Ajila C.M., Brar S.K., Verma M., Tyagi R.D., Godbout S., Valéro J.R. (2011). Extraction and Analysis of Polyphenols: Recent Trends. Crit. Rev. Biotechnol..

[105] Cuyckens F., Claeys M. (2004). Mass Spectrometry in the Structural Analysis of Flavonoids. J. Mass. Spectrom..

[106] Rocchetti G., Bhumireddy S.R., Giuberti G., Mandal R., Lucini L., Wishart D.S. (2019). Edible Nuts Deliver Polyphenols and Their Transformation Products to the Large Intestine: An In Vitro Fermentation Model Combining Targeted/Untargeted Metabolomics. Food Res. Int..

[107] Arribas A.S., Martínez-Fernández M., Chicharro M. (2012). The Role of Electroanalytical Techniques in Analysis of Polyphenols in Wine. TrAC Trends Anal. Chem..

[108] Arráez-Román D., Fu S., Sawalha S.M.S., Segura-Carretero A., Fernández-Gutiérrez A. (2010). HPLC/CE-ESI-TOF-MS Methods for the Characterization of Polyphenols in Almond-Skin Extracts. Electrophoresis.

[109] Tripathi S., Mazumder P.M. (2021). Cellular Investigations to Uncover Curative Potentials of Polyphenols—An In Vitro Study of Apple Cider Vinegar (ACV) and Chrysin against Alzheimer’s like Pathology via down-Regulation of AChE Activity. Indian J. Tradit. Knowl..

[110] Karthivashan G., Park S.Y., Kweon M.H., Kim J., Haque M.E., Cho D.Y., Kim I.S., Cho E.A., Ganesan P., Choi D.K. (2018). Ameliorative Potential of Desalted *Salicornia europaea* L. Extract in Multifaceted Alzheimer’s-like Scopolamine-Induced Amnesic Mice Model. Sci. Rep..

[111] Qiu W.Q., Pan R., Tang Y., Zhou X.G., Wu J.M., Yu L., Law B.Y.K., Ai W., Yu C.L., Qin D.L. (2020). Lychee Seed Polyphenol Inhibits Aβ-Induced Activation of NLRP3 Inflammasome via the LRP1/AMPK Mediated Autophagy Induction. Biomed. Pharmacother..

[112] Les F., Valero M.S., Moliner C., Weinkove D., López V., Gómez-Rincón C. (2021). *Jasonia glutinosa* (L.) Dc., a Traditional Herbal Tea, Exerts Antioxidant and Neuroprotective Properties in Different In Vitro and In Vivo Systems. Biology.

[113] Moliner C., Barros L., Dias M.I., Reigada I., Ferreira I.C.F.R., López V., Langa E., Rincón C.G. (2019). Viola Cornuta and Viola x Wittrockiana: Phenolic Compounds, Antioxidant and Neuroprotective Activities on Caenorhabditis Elegans. J. Food Drug Anal..

[114] Tan F.H.P., Najimudin N., Watanabe N., Shamsuddin S., Azzam G. (2023). P-Coumaric Acid Attenuates the Effects of Aβ42 In Vitro and in a Drosophila Alzheimer’s Disease Model. Behav. Brain Res..

[115] Leri M., Bertolini A., Stefani M., Bucciantini M. (2021). Evoo Polyphenols Relieve Synergistically Autophagy Dysregulation in a Cellular Model of Alzheimer’s Disease. Int. J. Mol. Sci..

[116] Leri M., Vasarri M., Carnemolla F., Oriente F., Cabaro S., Stio M., Degl’Innocenti D., Stefani M., Bucciantini M. (2023). EVOO Polyphenols Exert Anti-Inflammatory Effects on the Microglia Cell through TREM2 Signaling Pathway. Pharmaceuticals.

[117] Maiuolo J., Costanzo P., Masullo M., D’Errico A., Nasso R., Bonacci S., Mollace V., Oliverio M., Arcone R. (2023). Hydroxytyrosol–Donepezil Hybrids Play a Protective Role in an In Vitro Induced Alzheimer’s Disease Model and in Neuronal Differentiated Human SH-SY5Y Neuroblastoma Cells. Int. J. Mol. Sci..

[118] Maiuolo J., Bosco F., Guarnieri L., Nucera S., Ruga S., Oppedisano F., Tucci L., Muscoli C., Palma E., Giuffrè A.M. (2023). Protective Role of an Extract Waste Product from Citrus Bergamia in an In Vitro Model of Neurodegeneration. Plants.

[119] Taram F., Ignowski E., Duval N., Linseman D.A. (2018). Neuroprotection Comparison of Rosmarinic Acid and Carnosic Acid in Primary Cultures of Cerebellar Granule Neurons. Molecules.

[120] Xiao S., Lu Y., Wu Q., Yang J., Chen J., Zhong S., Eliezer D., Tan Q., Wu C. (2021). Fisetin Inhibits Tau Aggregation by Interacting with the Protein and Preventing the Formation of β-Strands. Int. J. Biol. Macromol..

[121] Hole K.L., Staniaszek L.E., Menon Balan G., Mason J.M., Brown J.T., Williams R.J. (2021). Oral (−)-Epicatechin Inhibits Progressive Tau Pathology in RTg4510 Mice Independent of Direct Actions at GSK3β. Front. Neurosci..

[122] Mahnashi M.H., Ashraf M., Alhasaniah A.H., Ullah H., Zeb A., Ghufran M., Fahad S., Ayaz M., Daglia M. (2023). Polyphenol-Enriched Desmodium Elegans DC. Ameliorate Scopolamine-Induced Amnesia in Animal Model of Alzheimer’s Disease: In Vitro, In Vivo and In Silico Approaches. Biomed. Pharmacother..

[123] Reutzel M., Grewal R., Silaidos C., Zotzel J., Marx S., Tretzel J., Eckert G.P. (2018). Effects of Long-Term Treatment with a Blend of Highly Purified Olive Secoiridoids on Cognition and Brain ATP Levels in Aged NMRI Mice. Oxid. Med. Cell Longev..

[124] Tai Y.-H., Lin Y.-Y., Wang K.-C., Chang C.-L., Chen R.-Y., Wu C.-C., Cheng I.H. (2018). Curcuminoid Submicron Particle Ameliorates Cognitive Deficits and Decreases Amyloid Pathology in Alzheimer’s Disease Mouse Model. Oncotarget.

[125] Kenchappa P.G., Karthik Y., Vijendra P.D., Hallur R.L.S., Khandagale A.S., Pandurangan A.K., Jayanna S.G., Alshehri M.A., Alasmari A., Sayed S. (2023). In Vitro Evaluation of the Neuroprotective Potential of Olea Dioica against Aβ Peptide-Induced Toxicity in Human Neuroblastoma SH-SY5Y Cells. Front. Pharmacol..

[126] El-Hawary S.S., Sobeh M., Badr W.K., Abdelfattah M.A.O., Ali Z.Y., El-Tantawy M.E., Rabeh M.A., Wink M. (2020). HPLC-PDA-MS/MS Profiling of Secondary Metabolites from Opuntia Ficus-Indica Cladode, Peel and Fruit Pulp Extracts and Their Antioxidant, Neuroprotective Effect in Rats with Aluminum Chloride Induced Neurotoxicity. Saudi J. Biol. Sci..

[127] Dostal V., Roberts C.M., Link C.D. (2010). Genetic Mechanisms of Coffee Extract Protection in a Caenorhabditis Elegans Model of β-Amyloid Peptide Toxicity. Genetics.

[128] Palmioli A., Mazzoni V., De Luigi A., Bruzzone C., Sala G., Colombo L., Bazzini C., Zoia C.P., Inserra M., Salmona M. (2022). Alzheimer’s Disease Prevention through Natural Compounds: Cell-Free, In Vitro, and In Vivo Dissection of Hop (*Humulus lupulus* L.) Multitarget Activity. ACS Chem. Neurosci..

[129] Iwata K., Wu Q., Ferdousi F., Sasaki K., Tominaga K., Uchida H., Arai Y., Szele F.G., Isoda H. (2020). Sugarcane (*Saccharum officinarum* L.) Top Extract Ameliorates Cognitive Decline in Senescence Model SAMP8 Mice: Modulation of Neural Development and Energy Metabolism. Front. Cell Dev. Biol..

[130] Eggers C., Fujitani M., Kato R., Smid S. (2019). Novel Cannabis Flavonoid, Cannflavin A Displays Both a Hormetic and Neuroprotective Profile against Amyloid β-Mediated Neurotoxicity in PC12 Cells: Comparison with Geranylated Flavonoids, Mimulone and Diplacone. Biochem. Pharmacol..

[131] Chethana K., Sasidhar B., Naika M., Keri R. (2018). Phytochemical Composition of Caesalpinia Crista Extract as Potential Source for Inhibiting Cholinesterase and β-Amyloid Aggregation: Significance to Alzheimer’s Disease. Asian Pac. J. Trop. Biomed..

[132] Kundo N.K., Manik M.I.N., Biswas K., Khatun R., Al-Amin M.Y., Alam A.H.M.K., Tanaka T., Sadik G. (2021). Identification of Polyphenolics from Loranthus Globosus as Potential Inhibitors of Cholinesterase and Oxidative Stress for Alzheimer’s Disease Treatment. Biomed. Res. Int..

[133] Ali A., Cottrell J.J., Dunshea F.R. (2022). Identification and Characterization of Anthocyanins and Non-Anthocyanin Phenolics from Australian Native Fruits and Their Antioxidant, Antidiabetic, and Anti-Alzheimer Potential. Food Res. Int..

[134] Pérez-Arribas L.V., León-González M.E., Rosales-Conrado N. (2017). Learning Principal Component Analysis by Using Data from Air Quality Networks. J. Chem. Educ..

[135] Sereia A.L., de Oliveira M.T., Baranoski A., Medeiros Marques L.L., Ribeiro F.M., Isolani R.G., de Medeiros D.C., Chierrito D., Lazarin-Bidóia D., Ferreira Zielinski A.A. (2019). In Vitro Evaluation of the Protective Effects of Plant Extracts against Amyloid-Beta Peptide-Induced Toxicity in Human Neuroblastoma SH-SY5Y Cells. PLoS ONE.

[136] Shabbir U., Tyagi A., Ham H.J., Elahi F., Oh D.H. (2022). Effect of Fermentation on the Bioactive Compounds of the Black Soybean and Their Anti-Alzheimer’s Activity. Front. Nutr..

[137] Shabbir U., Tyagi A., Ham H.J., Oh D.H. (2022). Comprehensive Profiling of Bioactive Compounds in Germinated Black Soybeans via UHPLC-ESI-QTOF-MS/MS and Their Anti-Alzheimer’s Activity. PLoS ONE.

[138] Yan N., Zhang H., Zhang Z., Zhang H., Zhou L., Chen T., Feng S., Ding C., Yuan M. (2022). The Extraction, Antioxidant and against β-Amyloid Induced Toxicity of Polyphenols from Alsophila Spinulosa Leaves. Arab. J. Chem..

[139] Francenia Santos-Sánchez N., Salas-Coronado R., Hernández-Carlos B., Villanueva-Cañongo C. (2019). Shikimic Acid Pathway in Biosynthesis of Phenolic Compounds. Plant Physiological Aspects of Phenolic Compounds.

[140] Mueed A., Shibli S., Al-Quwaie D.A., Ashkan M.F., Alharbi M., Alanazi H., Binothman N., Aljadani M., Majrashi K.A., Huwaikem M. (2023). Extraction, Characterization of Polyphenols from Certain Medicinal Plants and Evaluation of Their Antioxidant, Antitumor, Antidiabetic, Antimicrobial Properties, and Potential Use in Human Nutrition. Front. Nutr..

[141] Teigiserova D.A., Hamelin L., Thomsen M. (2020). Towards Transparent Valorization of Food Surplus, Waste and Loss: Clarifying Definitions, Food Waste Hierarchy, and Role in the Circular Economy. Sci. Total Environ..

[142] Crespo L., Sede Lucena B., Martínez F.G., Mozzi F., Pescuma M. (2024). Selenium Bioactive Compounds Produced by Beneficial Microbes. Adv. Appl. Microbiol..

[143] Kabir E.R., Chowdhury N.M., Yasmin H., Kabir M.T., Akter R., Perveen A., Ashraf G.M., Akter S., Rahman M.H., Sweilam S.H. (2023). Unveiling the Potential of Polyphenols as Anti-Amyloid Molecules in Alzheimer’s Disease. Curr. Neuropharmacol..

[144] El Gaamouch F., Chen F., Ho L., Lin H.-Y., Yuan C., Wong J., Wang J. (2022). Benefits of Dietary Polyphenols in Alzheimer’s Disease. Front. Aging Neurosci..

[145] Jabir N.R., Khan F.R., Tabrez S. (2018). Cholinesterase Targeting by Polyphenols: A Therapeutic Approach for the Treatment of Alzheimer’s Disease. CNS Neurosci. Ther..

[146] Nasso R., Pagliara V., D’Angelo S., Rullo R., Masullo M., Arcone R. (2021). Annurca Apple Polyphenol Extract Affects Acetyl- Cholinesterase and Mono-Amine Oxidase In Vitro Enzyme Activity. Pharmaceuticals.

[147] Shahabadi N., Zendehcheshm S., Khademi F. (2022). Green Synthesis, In Vitro Cytotoxicity, Antioxidant Activity and Interaction Studies of CuO Nanoparticles with DNA, Serum Albumin, Hemoglobin and Lysozyme. ChemistrySelect.

[148] Al-Radadi N.S. (2022). Single-Step Green Synthesis of Gold Conjugated Polyphenol Nanoparticle Using Extracts of Saudi’s Myrrh: Their Characterization, Molecular Docking and Essential Biological Applications. Saudi Pharm. J..

[149] Thatyana M., Dube N.P., Kemboi D., Manicum A.-L.E., Mokgalaka-Fleischmann N.S., Tembu J.V. (2023). Advances in Phytonanotechnology: A Plant-Mediated Green Synthesis of Metal Nanoparticles Using Phyllanthus Plant Extracts and Their Antimicrobial and Anticancer Applications. Nanomaterials.

[150] Slanzi A., Iannoto G., Rossi B., Zenaro E., Constantin G. (2020). In Vitro Models of Neurodegenerative Diseases. Front. Cell Dev. Biol..

[151] Sheeler C., Rosa J.-G., Ferro A., McAdams B., Borgenheimer E., Cvetanovic M. (2020). Glia in Neurodegeneration: The Housekeeper, the Defender and the Perpetrator. Int. J. Mol. Sci..

[152] Jávega B., Herrera G., Martínez-Romero A., O’Connor J.-E. (2023). Flow Cytometry of Oxygen and Oxygen-Related Cellular Stress. Oxygen.

[153] Rudrapal M., Khairnar S.J., Khan J., Dukhyil A.B., Ansari M.A., Alomary M.N., Alshabrmi F.M., Palai S., Deb P.K., Devi R. (2022). Dietary Polyphenols and Their Role in Oxidative Stress-Induced Human Diseases: Insights Into Protective Effects, Antioxidant Potentials and Mechanism(s) of Action. Front. Pharmacol..

[154] Al Mamun A., Shao C., Geng P., Wang S., Xiao J. (2024). Polyphenols Targeting NF-ΚB Pathway in Neurological Disorders: What We Know So Far?. Int. J. Biol. Sci..

[155] Álvarez-Berbel I., Espargaró A., Viayna A., Caballero A.B., Busquets M.A., Gámez P., Luque F.J., Sabaté R. (2022). Three to Tango: Inhibitory Effect of Quercetin and Apigenin on Acetylcholinesterase, Amyloid-β Aggregation and Acetylcholinesterase-Amyloid Interaction. Pharmaceutics.

[156] Andrade V., Cortés N., Pastor G., Gonzalez A., Ramos-Escobar N., Pastene E., Rojo L.E., MacCioni R.B. (2020). N-Acetyl Cysteine and Catechin-Derived Polyphenols: A Path Toward Multi-Target Compounds against Alzheimer’s Disease. J. Alzheimer’s Dis..

[157] Choi E.Y., Kang S.S., Lee S.K., Han B.H. (2020). Polyphenolic Biflavonoids Inhibit Amyloid-Beta Fibrillation and Disaggregate Preformed Amyloid-Beta Fibrils. Biomol. Ther..

[158] Ayyalasomayajula N., Ajumeera R., Chellu C.S., Challa S. (2019). Mitigative Effects of Epigallocatechin Gallate in Terms of Diminishing Apoptosis and Oxidative Stress Generated by the Combination of Lead and Amyloid Peptides in Human Neuronal Cells. J. Biochem. Mol. Toxicol..

[159] Aranda-Abreu G.E., Martínez-Díaz J.A., Hernández-Aguilar M.E., Rojas-Durán F., Herrera-Covarrubias D., García-Hernández L.I., Mestizo-Gutiérrez S.L. (2020). Expression of Proteins Linked to Alzheimer’s Disease in C6 Rat Glioma Cells under the Action of Lipopolysaccharide (LPS), Nimesulide, Resveratrol and Citalopram. Turk. J. Biochem..

[160] Chen P., Chen F., Lei J., Zhou B. (2023). Pomegranate Polyphenol Punicalagin Improves Learning Memory Deficits, Redox Homeostasis, and Neuroinflammation in Aging Mice. Phytother. Res..

[161] Hu Y., Hu X., Lu Y., Shi S., Yang D., Yao T. (2020). New Strategy for Reducing Tau Aggregation Cytologically by A Hairpinlike Molecular Inhibitor, Tannic Acid Encapsulated in Liposome. ACS Chem. Neurosci..

[162] Mattioli R., Francioso A., D’Erme M., Trovato M., Mancini P., Piacentini L., Casale A.M., Wessjohann L., Gazzino R., Costantino P. (2019). Anti-Inflammatory Activity of a Polyphenolic Extract from Arabidopsis Thaliana in In Vitro and In Vivo Models of Alzheimer’s Disease. Int. J. Mol. Sci..

[163] Hase T., Shishido S., Yamamoto S., Yamashita R., Nukima H., Taira S., Toyoda T., Abe K., Hamaguchi T., Ono K. (2019). Rosmarinic Acid Suppresses Alzheimer’s Disease Development by Reducing Amyloid β Aggregation by Increasing Monoamine Secretion. Sci. Rep..

[164] Yang L., Cui Y., Liang H., Li Z., Wang N., Wang Y., Zheng G. (2022). Multifunctional Selenium Nanoparticles with Different Surface Modifications Ameliorate Neuroinflammation through the Gut Microbiota-NLRP3 Inflammasome-Brain Axis in APP/PS1 Mice. ACS Appl. Mater. Interfaces.

[165] Tomaselli S., La Vitola P., Pagano K., Brandi E., Santamaria G., Galante D., D’Arrigo C., Moni L., Lambruschini C., Banfi L. (2019). Biophysical and In Vivo Studies Identify a New Natural-Based Polyphenol, Counteracting Aβ Oligomerization In Vitro and Aβ Oligomer-Mediated Memory Impairment and Neuroinflammation in an Acute Mouse Model of Alzheimer’s Disease. ACS Chem. Neurosci..

[166] Bai X., Liu C.M., Li H.J., Zhang Z.P., Cui W.B., An F.L., Zhang Z.X., Wang D.S., Fei D.Q. (2023). Ethyl Caffeate Attenuates Aβ-Induced Toxicity in Caenorhabditis Elegans AD Models via the Insulin/Insulin-like Growth Factor-1 Signaling Pathway. Bioorg Chem..

[167] Prüßing K., Voigt A., Schulz J.B. (2013). Drosophila Melanogaster as a Model Organism for Alzheimer’s Disease. Mol. Neurodegener..

[168] Sonawane S.K., Uversky V.N., Chinnathambi S. (2021). Baicalein Inhibits Heparin-Induced Tau Aggregation by Initializing Non-Toxic Tau Oligomer Formation. Cell Commun. Signal..

[169] Puzzo D., Lee L., Palmeri A., Calabrese G., Arancio O. (2014). Behavioral Assays with Mouse Models of Alzheimer’s Disease: Practical Considerations and Guidelines. Biochem. Pharmacol..

[170] Schmid S., Rammes G., Blobner M., Kellermann K., Bratke S., Fendl D., Kaichuan Z., Schneider G., Jungwirth B. (2019). Cognitive Decline in Tg2576 Mice Shows Sex-Specific Differences and Correlates with Cerebral Amyloid-Beta. Behav. Brain Res..

